# Dynamics and Molecular Determinants of Cytoplasmic Lipid Droplet Clustering and Dispersion

**DOI:** 10.1371/journal.pone.0066837

**Published:** 2013-06-25

**Authors:** David J. Orlicky, Jenifer Monks, Adrianne L. Stefanski, James L. McManaman

**Affiliations:** 1 Department of Pathology, University of Colorado School of Medicine, Aurora, Colorado, United States of America; 2 Division of Basic Reproductive Sciences, Department of Obstetrics and Gynecology, University of Colorado School of Medicine, Aurora, Colorado, United States of America; 3 Graduate Program in Reproductive Sciences, University of Colorado School of Medicine, Aurora, Colorado, United States of America; The University of Queensland, Australia

## Abstract

Perilipin-1 (Plin1), a prominent cytoplasmic lipid droplet (CLD) binding phosphoprotein and key physiological regulator of triglyceride storage and lipolysis in adipocytes, is thought to regulate the fragmentation and dispersion of CLD that occurs in response to β-adrenergic activation of adenylate cyclase. Here we investigate the dynamics and molecular determinants of these processes using cell lines stably expressing recombinant forms of Plin1 and/or other members of the perilipin family. Plin1 and a C-terminal CLD-binding fragment of Plin1 (Plin1CT) induced formation of single dense CLD clusters near the microtubule organizing center, whereas neither an N-terminal CLD-binding fragment of Plin1, nor Plin2 or Plin3 induced clustering. Clustered CLD coated by Plin1, or Plin1CT, dispersed in response to isoproterenol, or other agents that activate adenylate cyclase, in a process inhibited by the protein kinase A inhibitor, H89, and blocked by microtubule disruption. Isoproterenol-stimulated phosphorylation of CLD-associated Plin1 on serine 492 preceded their dispersion, and live cell imaging showed that cluster dispersion involved initial fragmentation of tight clusters into multiple smaller clusters, which then fragmented into well-dispersed individual CLD. siRNA knockdown of the cortical actin binding protein, moesin, induced disaggregation of tight clusters into multiple smaller clusters, and inhibited the reaggregation of dispersed CLD into tight clusters. Together these data suggest that the clustering and dispersion processes involve a complex orchestration of phosphorylation-dependent, microtubule-dependent and independent, and microfilament dependent steps.

## Introduction

Cytoplasmic lipid droplets (CLD) are organelle-like structures that function in the storage and transfer of neutral lipids for use as a source of energy, for membrane synthesis, and for production of bioactive signaling molecules [1]. To accomplish these functions, CLD move along a network of microtubules to deliver lipid substrates within the cell. Microtubule depolymerization inhibits CLD movement in a number of systems [2], [3], and microtubule-associated proteins including tubulin and the microtubule motors, dynein, and kinesin, have been identified on CLD by proteomic [4], [5] and genetic screens [6]. However, questions remain about the mechanisms governing CLD interactions with microtubules, and how the direction and destination of CLD movement are specified [7]. Although elements of the actin-filament system have also been identified on CLD [8], [9], disrupting actin filaments does not appear to prevent movement of stomatin-coated CLD [10] suggesting that actin-based transport does not directly contribute to CLD transport.

Members of the perilipin (PLIN) family of CLD-binding proteins are known to influence formation and maturation of CLD [11]–[13], and there is increasing evidence that PLIN family members function in trafficking and specifying the cellular itineraries of CLD, and in determining interactions between individual CLD and/or between CLD and other subcellular structures [14]–[16]. For example, lipid storage droplet 2 (LSD-2), a *Drosophila* homologue of Plin1, mediates CLD transport during *Drosophila* oogenesis [17], and perilipin2 (Plin2/adipophilin/ADRP) is reported to maintain the dispersed distribution of CLD in hepatitis C virus infected HUH7 cells [14]. Perilipin (Plin1) on the other hand is implicated in both clustering and dispersion of CLD in fibroblasts and HEK293 cells [15], [16], [18]. Furthermore, when the effects of ectopically expressed Plin1, Plin2 and Plin3 (TIP47) on CLD distribution in HEK293 cells were directly compared, only Plin1 directed clustering [18]. These observations implicate Plin1 as a specific determinant of interactions that promote aggregation and clustering of CLD and, by extension, possibly their motility and cellular localization.

Observations that protein kinase A (PKA)-dependent phosphorylation of Plin1 induces dispersion of clustered CLD in fibroblasts and 3T3L1 adipocytes [5], [15] suggest that the phosphorylation state of Plin1 may govern interactions between CLD and microtubules. However, it is unknown if Plin1-phosphorylation directly recruits microtubule motors to CLD, or if it leads to the recruitment of adaptor proteins that mediate CLD-microtubule interactions [15]. For instance, isoproterenol-stimulated dispersion not only induces Plin1 phosphorylation, it is also known to induce the localization of Plin2 to the CLD surface [19], raising the possibility that interactions between Plin2 and microtubules may mediate phosphorylation-dependent dispersion of CLD. Central to the Plin1 phosphorylation issue, a careful analysis of dispersion and Plin1 phosphorylation in the same cell has not yet been reported.

In this study we use HEK293 cells stably expressing native and mutant forms of Plin1, as well as Plin2 and Plin3 to investigate the mechanisms regulating CLD clustering and dispersion. Our results show that the C-terminal region of Plin1 mediates CLD clustering and dispersion; that CLD cluster near the microtubule organizer center (MTOC); and that clustering is dependent on microtubules and the actin-associated protein, moesin. We further show that dispersion requires an intact microtubule network, and occurs by a complex process mediated by both phosphorylation-dependent and phosphorylation-independent steps. We also show the temporal relationship between phosphorylation of Plin1 and dispersion.

## Experimental Procedures

### Materials

Reagents were purchased from Sigma Chemical Company (St. Louis, MO) unless otherwise indicated. Triacsin C was purchased from BioMol (Plymouth Meeting, PA), H-89 was purchased from InvivoGen (San Diego, CA), and 8-(4-Chlorophenylthio)-2′-O-methyladenosine-3′,5′-cyclic monophosphate was purchased from Tocris Bioscience (Bristol, UK). The following types and sources of antibodies were used in our study: Guinea pig antibodies to mouse Plin 1 (Fitzgerald Inc, Concord, MA); mouse monoclonal antibodies to human Plin1 phosphorylated on Ser497 (Vala Sciences, San Diego); chicken antibodies to human TIP47 (Genway, San Diego, CA); mouse monoclonal anti-gamma-tubulin and rabbit polyclonal anti-beta tubulin (Novus Biologicals, Littleton, CO), chicken polyclonal anti-beta-actin and rabbit polyclonal antibodies to moesin, ezrin, and the 5A, 5B, and 5C isoforms of kinesin (Abcam, Cambridge, MA); mouse monoclonal anti-GM130 (BD Biosciences, Indianapolis, IN); rabbit polyclonal anti-calreticulin (StressGen Biotechnologies Corp, Victoria, BC Canada); mouse monoclonal anti-dynein (Thermo Scientific (Rockford, IL), and mouse monoclonal anti-VSV-G, (11-amino-acid epitope; Roche, Indianapolis, IN; clone P5D4). The mouse monoclonal anti-LAMP2 antibody developed by J. Thomas August (John Hopkins University, Baltimore, MD) was obtained from the Developmental Studies Hybridoma Bank developed under the auspices of the NICHD and maintained by The University of Iowa, Department of Biology, Iowa City, IA.

### Cell Culture

HEK293 cells express Plin3 endogenously but do not express Plin2 or Plin1 [20]. HEK293 cell lines stably expressing mouse Plin2-VSV, mouse Plin1, perilipin-GFP (Plin1-GFP) and mouse Plin3-VSV (TIP47-VSV) or co-expressing Plin2-VSV and Plin1 (Plin1-Plin2) have been described previously [18], [20]. The amino acid sequence used to epitope tag Plin2 and Plin3 is the VSV-G sequence (encoded by the amino acid sequence YTDIEMNRLGK, Roche Diagnostics, Indianapolis, IN), hereafter referred to as VSV. HEK293 cells expressing Plin1 amino acids 1–197 or 198–517 were selected from cultures transfected with pcDNA3 plasmids encoding that portion of the Plin1 protein (generated by PCR deletion mutagenesis) and C-terminally tagged with the VSV sequence to generate Plin1(1–197)-VSV and Plin1(198–517)-VSV cell lines respectively. Clones from all of these cell lines were cultured in DMEM medium supplemented with 6% fetal calf serum (control media, CM) or CM supplemented with 100 µM oleic acid (CM+OA), for 24 hours. Penicillin and streptomycin were not added to the culture medium. Stock stably transfected cell lines were maintained in G418, however all experiments were conducted in CM in its absence. Only data from twice-cloned cell lines are presented. Cultured cells were grown on glass cover slips in culture dishes for at least 3 days in control media prior to experimentation. Isoproterenol was reconstituted in water and added to cultures to a final concentration of 10 µg/ml. Forskolin was reconstituted in DMSO at 10 mM and used at a final concentration of 10 µM. Nocodazole was reconstituted in DMSO at 0.2 mg/ml and was added to cultures at a final concentration of 0.2 µg/ml. The length of time of incubation with isoproterenol, forskolin or nocodazole is indicated in the text. DMSO diluted 1/1000 was used as the vehicle control for forskolin or nocodazole experiments. Cells were incubated in media containing 8 µM triacsin C to inhibit TAG synthesis [20], [21], 1 mM aminoimidazole carboxamide ribonucleotide (AICAR) to stimulate AMP-activated protein kinase (AMPK) and 20 µM H-89 to inhibit PKA-dependent phosphorylation of Plin1. Triacsin C activity was verified as described previously by preventing CLD formation [20]. AICAR activity was varified by AMPK phosphorylation [22], using phospho-AMPK antibodies (Cell Signaling). H-89 inhibition of mouse Plin1 phosphorylation on S492 was verified using mouse monoclonal antibodies to human phospho-Ser497 Plin1 (Vala Sciences), which also recognize mouse phospho-Ser492 Plin1.

Following experimental treatments, media was removed and the cells were fixed with 3.7% formaldehyde for 10 minutes, permeabilized with 50% ethanol or 0.05% triton X-100, washed with phosphate buffered saline for at least 30 minutes, and immunohistochemically stained as previously described [20]. Cells were also fixed in methanol at −20°C, in 30% methanol plus 70% acetone at −20°C, or in 1% electron microscopy grade glutaraldehyde at 4°C to corroborate localization results utilizing 3.7% formaldehyde as the fixative. In all experiments involving staining of moesin and ezrin, fixed cells were permeabilized with 0.05% triton X-100.

### siRNA Experiments

Cells were plated on glass cover slips and 24 hours later were transfected with 10 nM siRNA (Ambion Inc., Carlsbad, CA) using Lipofectamine RNAiMAX (Invitrogen, Carlsbad, CA) according to the manufacturers instructions. After 24 hours, media was changed and then again after 48 hours. siRNA experiments were terminated at 72 hours. siRNAs used for ezrin, moesin, and Plin3 knockdown experiments (Table 1) were validated to decrease their respective mRNA by >95% at 48 hours post transfection by the manufacturer.

**Table 1:** Sequence of siRNA oligonucleotides.

	Plin3
sense	UAUUCGCUGGCUGAUGCAAUCUGGG
antisense	CCCAGAUUGCAUCAGCCAGCGAAUA
	**Moesin**
sense	GGUGUGAACUACUUCAGCATT
antisense	UGCUGAAGUAGUUCACACCAT
	**Ezrin**
Sense	GGAAUCAACUAUUUCGAGATT
antisense	UCUCGAAAUAGUUGAUUCCAT

### Immunoblot Analysis

Cells were lysed and soluble proteins were separated by SDS-PAGE on 10% polyacrylamide gels and transferred to nitrocellulose membranes as described previously [20]. Plin1 was detected by probing the membranes with guinea pig anti-perilipin (1∶5000). Immunoreactive bands were detected using horseradish peroxidase conjugated secondary antibody (Sigma Chemical Co., St. Louis, MO) and Western Lightning Chemiluminescence Reagent (Perkin Elmer, Boston, MA). Duplicate dishes were assayed for each data point and all experiments were done at least 3 times.

### Image Analysis and CLD Quantitation

Immunofluorescence images of fixed cells were captured at room temperature on a Nikon Diaphot fluorescence microscope equipped with a Cooke SensiCam CCD camera (Tonawand, NY) using Slidebook software (Intelligent Imaging Innovations Inc., Denver CO) as previously described [20]. Fluorescence images were digitally deconvolved using the No Neighbors algorithm (Slidebook) and converted to TIFF files. All images were processed and assembled into montages in Photoshop (Adobe Systems Inc. Mountain View, CA). Images shown in any one montage have been taken and processed similarly to allow visual comparisons of relative protein levels in cells expressing the same protein(s).

Quantitation of the dispersion of CLD clusters was performed in two ways. First, the stage of dispersion of CLD was performed on 600x magnification images of randomly selected fields of cells, by an investigator blinded to the treatments. CLD were classified into three stages of dispersion based on previously described morphological criteria in cultured fibroblasts [15]: single tight CLD clusters (Stage 1); multiple partially disaggregated clusters (Stage 2); or completely dispersed individual CLD (Stage 3). An average of 50–100 cells per group was analyzed for their stage for each experimental condition for each of at least three separate experiments. Secondly, No Neighbors deconvolved images of fixed cells were segmented in SlideBook software using the Ridley Calvert segmentation method, to count nuclei and to count the total number of perilipin-stained objects in the field. These segmentations were used to calculate the number of objects per field as an unbiased measure of CLD dispersion. Experiments were performed a minimum of three times. Data presented is the combined data from all experiments.

Quantitation of perilipin phosphorylation was performed on cells co-stained with anti-mouse Plin1 and anti-human Plin1 phosphorylated on S497. No Neighbors deconvolved images were segmented for phospho-Plin1 selecting all data above a minimum background, equally in all conditions. The area of the mask was then compared to the total Plin1 staining to determine the fraction phosphorylated. Experiments were performed a minimum of three times. Data presented is the combined data from all experiments.

Detection of CLD protein co-localization was measured using Slidebook software which quantified the number of pixels with overlapping fluorescence. Cross channel Pearson’s correlations were then performed using Slidebook software.

### Live Cell Imaging

HEK293 cells, stably transfected with GFP-Plin1 were plated in special glass bottom culture dishes (MatTek Corporation, Ashland, MA; Catalog Number P35G-0-20-C) and allowed to grow for 3 days. The evening prior to imaging, media was replaced with phenol red-free DMEM medium to which was added 10 mM HEPES buffer (pH 7.35). Dishes were then placed on the prewarmed stage of an inverted florescence microscope in an environmental chamber maintained at 37°C and 5% CO_2_ and imaged every 1 second to 4 minutes for up to 1 hour. Z-series images were collected at each time point using an Olympus IX81 fluorescence microscope equipped with a cooled Hamamatsu CCD video camera. Projection images of each Z-series were generated using Slidebook Software.

Live-imaging was captured on a 3I Marianas inverted Spinning Disk system built on a Zeiss Axio Observer Z1 with Yokogawa CSU-X1 spinning disk, a Photometrics Evolve 16-bit EMCCD camera, millisecond 3I mSwitcher, for rapid multicolor imaging, 3I mSAC millisecond spherical aberation correction, Okolab cage incubator with temperature, CO_2_, air and humidity control, and 3I Slidebook 5.0 software. The system was built by Intelligent Imaging Innovations, Inc. (Denver, CO) and maintained in the Advanced Light Microscopy Facility of the University of Colorado, Anschutz Medical Campus.

### Data Analysis

Two-tailed unpaired Student’s T-tests were performed to evaluate differences between Pearson’s correlations using GraphPad Prism version 5.0d for Mac OS X. Chi-square analyses were performed on the summed averages of percentages of CLD in each stage using GraphPad Prism version 5.0d for Mac OS X. One-way ANOVA with Tukey’s post test analysis was used to examine differences between groups following quantitation of the number of objects/cell using GraphPad Prism version 5.0a for Mac OS X. Where applicable a one-way ANOVA with a post-test for linear trends was performed with GraphPad Prism.

## Results

### Plin Family Members Differentially Affect CLD Localization and Dispersion in Response to Isoproterenol Stimulation

**Figure 1:**
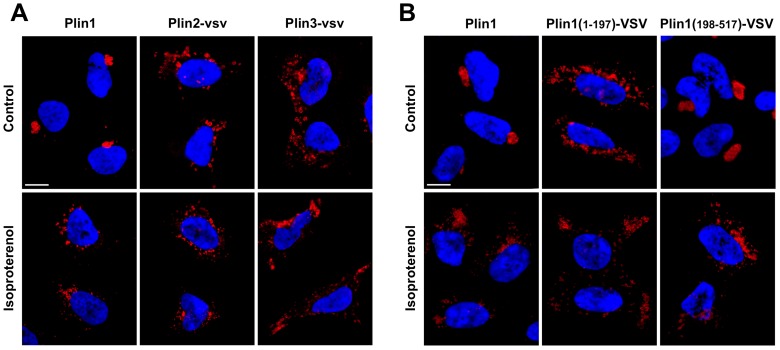
Plin family members and isoproterenol exposure determine CLD distribution within HEK293 cells. (A) Representative images of CLD localization in HEK293 cells stably expressing Plin1, Plin2-VSV or Plin3-VSV and incubated in culture media without (Control) or with 10 µg/ml isoproterenol for 1 hour (Isoproterenol). CLD in cells expressing Plin1 were immunolocalized with antibodies to Plin1. CLD in cells expressing Plin2-VSV or Plin3-VSV were immunolocalized with antibodies to VSV. Cells expressing Plin1 were cultured in CM. Cells expressing Plin2-VSV or Plin3-VSV were cultured in CM+OA. Fluorescence of Hoechst-stained nuclei (N) is included for orientation. The size bar is 10 µm. (B) Effects of N- and C-terminal regions of Plin1 on CLD distribution. Representative images are shown of CLD localization in HEK293 cells stably expressing full length Plin1, Plin1(1–197)-VSV or Plin1(198–517)-VSV following incubation in media without (Control) or with 10 µg/ml isoproterenol for 1 hour (Isoproterenol). CLD in Plin1-expressing cells were immunolocalized with antibodies to Plin1, those in cells expressing peri(1–197)-VSV or peri(198–518)-VSV were immunolocalized with antibodies to VSV. Cells expressing Plin1 or peri(198–518)-VSV were cultured in CM. Cells expressing peri(1–197)-VSV were cultured in CM+OA. Fluorescence of Hoechst-stained nuclei (N) is included for orientation. The size bar is 10 µm.

Ectopic Plin1 expression in fibroblasts and HEK293 cells is known to induce the accumulation and clustering of CLD [15], [16], [18], and PKA-dependent phosphorylation of mouse Plin1 on Ser492 is known to be required for disaggregation and dispersion of clustered CLD in response to agents that increase intracellular cAMP levels [15]. To better understand the mechanisms by which CLD clustering and dispersion are regulated, and to assess possible contributions of other perilipin family members to these processes, we utilized HEK293-derived cell lines that stably express Plin1 or Plin2 ectopically [18]. As reported previously [18], in HEK293 cells expressing Plin1 (Plin1 cells), densely clustered Plin1-positive CLD accumulate near one pole of the nucleus when cultured in control media (CM) (Figure 1A). Consistent with findings from experiments in which Plin1 was ectopically expressed in fibroblasts [15], we found that CLD clusters within these cells were completely dispersed following exposure to 10 µg/ml isoproterenol for 1 hour. Furthermore, we found that isoproterenol-induced cluster dispersion correlated with phosphorylation of Plin1 as detected by an up-shift in the Plin1 band on SDS PAGE (Figure S1). Thus, the general effects of ectopically expressed Plin1 on CLD clustering and dispersion in HEK293 cells appear to be similar to those of ectopically expressed Plin1 in fibroblasts (15).

To determine if Plin1 was unique among perilipin family members in directing the clustering of CLD, Plin2 and Plin3 were expressed in HEK293 cells as C-terminal VSV-G epitope tagged proteins (Plin2-VSV or Plin3-VSV). As previously reported [18], we did not detect CLD in cells expressing these constructs unless they were cultured in media supplemented with oleic acid (CM+OA) to stimulate triglyceride (TAG) synthesis. Under these conditions, CLD coated by Plin2-VSV or Plin3-VSV were dispersed widely within the cytoplasm (Figure 1A), and isoproterenol treatment did not appear to affect the degree of their dispersion or alter their cellular distribution. Thus, in contrast to Plin1, neither Plin2 nor Plin3 induced tight CLD clustering, and the distribution of CLD coated by these proteins did not appear to be significantly influenced by isoproterenol.

### The C-terminal Region (CTR) of Plin1 Regulates CLD Clustering and Dispersion

Since Plin1 appears to direct both clustering and dispersion of CLD, we next sought to identify the functional determinants of its actions. Previous studies demonstrated that expression of C-terminal regions of Plin1 in fibroblasts induce CLD clustering [16], and that dispersion of clustered Plin1-coated CLD depends on PKA-dependent phosphorylation of Plin1 on S492 [15]. However, evidence that phosphorylation of serines within the Plin1 N-terminal region (NTR) alter the conformational properties of the Plin1 CTR [23] raise the possibility of NTR interactions contributing to clustering and/or hormone-dependent dispersion of Plin1 CLD. To further define the role of the Plin1 CTR in mediating clustering and dispersion of CLD we generated cell lines stably expressing N-terminal (amino acids 1–197) and C-terminal (amino acids 198–517) fragments of Plin1 as VSV-G epitope tagged proteins in HEK293 cells. Both portions of Plin1 were expected to bind CLD based on published evidence [16]. In cells expressing the 1–197 fragment, (Plin1(1–197)-VSV), the formation of CLD depended on culturing them in CM+OA. Under these conditions CLD coated by Plin1(1–197)-VSV were fully dispersed within the cytoplasm (Figure 1B), and isoproterenol exposure did not further affect their distribution. In contrast, in cells expressing the 198–517 fragment (Plin1(198–517)-VSV), we found tight perinuclear clusters of VSV-positive CLD when they were cultured in either CM (Figure 1B) or in CM+OA (data not shown). After exposure to isoproterenol for 1 hour, VSV-positive CLD in Plin1(198–517)-VSV cells were dispersed within the cytoplasm, indicating that the Plin1 CTR is sufficient for clustering and hormone-dependent dispersion of CLD.

### Plin2 and Plin3 do not Contribute to the Clustering or Dispersion of Plin1-coated CLD

Isoproterenol exposure has been shown to induce the association of Plin2 with Plin1-coated CLD in NIH-3T3 C/EBPα cells [19]. Because Plin2 is proposed to maintain CLD in a dispersed state [14], we next investigated whether the binding of Plin2 and/or possibly Plin3 to Plin1-coated CLD trigger their dispersion. The effects of Plin2 on Plin1-CLD dispersion were investigated in HEK293 cells that stably co-express Plin2-VSV and Plin1 (Plin2-Plin1 cells) [18]. When these cells were cultured in CM, we detected single dense CLD clusters near the nucleus that immunostained for Plin1 but not for Plin2-VSV (Figure S2). However, when Plin2-Plin1 cells were incubated with isoproterenol for 1 hour to induce CLD dispersion, we found that both Plin1 and Plin2-VSV were associated with dispersed CLD. To determine if the presence of Plin2-VSV on Plin1-coated CLD is sufficient to induce their dispersion, we incubated Plin2-Plin1 cells with CM supplemented with 100 µM oleic acid (CM+OA) to induce formation of CLD with both Plin2-VSV and Plin1 on their surface [18]. As shown in Figure S2, under these conditions we found tightly clustered perinuclear CLD that were positive for both Plin1 and Plin2-VSV in the absence of isoproterenol treatment. Upon addition of isoproterenol, these clusters underwent dispersion, and the dispersed CLD were also positive for both Plin1 and Plin2-VSV. Thus, Plin2 does not induce formation of tight CLD clusters on its own, nor does it mediate cluster dispersion.

We next determined if Plin3 influenced the clustering or dispersion of Plin1-coated CLD. While HEK293 cells endogenously express Plin3, Plin3 is not detected on Plin1-positive CLD unless cells are cultured in CM+OA [18] (Figure S3). We used siRNA oligonucleotides to reduce endogenous Plin3 expression in Plin1 cells (Figure S3). In Plin1 cells transfected with scrambled siRNA and cultured in CM+OA in the absence of isoproterenol, we found that CLD formed tight clusters that were positive for both Plin1 and Plin3. In Plin1 cells transfected with siRNA directed against Plin3, we again found tightly clustered CLD that were positive for Plin1, however these CLD clusters were not positive for Plin3, which validates that anti-Plin3 siRNA treatment effectively reduces endogenous Plin3 below immunodetection levels, and suggests that CLD clustering is not influenced by the presence or absence of Plin3. We also found that in Plin1 cells transfected with scrambled or Plin3 siRNA, isoproterenol treatment for 1 hour induced comparable degrees of CLD dispersion (Figure S3). These data suggest that, like Plin2, Plin3 does not contribute to clustering or dispersion of Plin1 coated CLD.

### Dispersion of Plin1 Coated CLD Occurs in Stages

**Figure 2:**
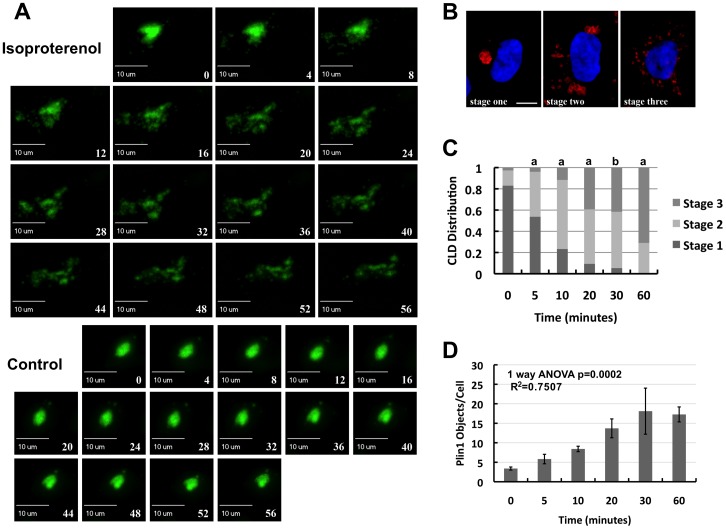
Time course and stages of CLD cluster dispersion. (A) Real-time dispersion of clusters coated with GFP-Plin1. CLD distribution in GFP-Plin1 expressing cells is shown at 4-minute intervals after their exposure to 10 µg/ml isoproterenol (Isoproterenol) or an equivalent volume of vehicle (Control). Scale bars and the time, in minutes, after isoproterenol exposure is shown in each panel. See Figure S4 for a movie of this time series. (B) Representative immunofluorescence images showing the three morphologically distinct stages of Plin1-coated CLD dispersion following exposure of cells to isoproterenol (10 µg/ml). Plin1-coated CLD are shown in red, Hoechst-stained nuclei are shown in blue. The size bar is 10 µm. (C) The change in CLD clustering as a function of time after exposure to isoproterenol monitored by morphological analysis. Data shown are averages of 5 experiments; in each experiment 60–100 cells were assayed per time point. Statistical significance is indicated by lower case letters: a, stage values are different from values at previous time points (p<0.001); b, values are different from 0, 5, and 10 minute values (p<0.001). (D) The change in CLD clustering as a function of time after exposure to isoproterenol monitored by Plin1 objects/cell. The values are means ± SEM for 5 experiments performed in duplicate. A 1-way ANOVA analysis of dispersion yields a p = 0.0002; the post test for linear trends p<0.0001, and R^2^ = 0.7507.

To better understand the process of CLD cluster dispersion we used live-cell imaging of HEK293 cells expressing GFP-Plin1. The Isoproterenol panels in Figure 2A show consecutive images at 4 minute intervals of a GFP-Plin1 expressing cell treated with isoproterenol. These panels demonstrate a single closely packed cluster of CLD that begins to loosen and to break into smaller clusters (decluster) approximately 4–8 minutes after isoproterenol addition. After 16–20 minutes, discrete smaller clusters are detectable, which subsequently disperse into yet smaller CLD clusters and individual CLD. In contrast, CLD remain tightly clustered in control treated cells over the same period (Figure 2A, control panels). This result is consistent with a step-wise process; tightly clustered CLD first decluster or fragment into smaller clusters before fully dispersing into separate CLD as observed in fibroblasts ectopically expressing Plin1 [15]. A real-time movie showing larger well-defined images of this full process is included in Figure S4.

We used two quantitative approaches to define the processes controlling Plin1-CLD dispersion. First, we classified isoproterenol-induced CLD dispersion according to morphological criteria that were previously used to describe the dispersion of Plin1-coated CLD in fibroblasts [15]. We found that in Plin1 cells, CLD also existed as single tight clusters (Stage 1), multiple, partially disaggregated, clusters (Stage 2); and individual, fully dispersed, CLD (Stage 3) (illustrated in Figure 2B). We used this approach to describe the temporal effects of isoproterenol on cluster morphology during dispersion (Figure 2C). In the non-stimulated condition (control) approximately 82% of the cells had CLD in Stage 1, 16% in Stage 2 and 2% in Stage 3. This distribution changed rapidly following isoproterenol addition, with a progressive decrease in the percentage of cells with CLD in Stage 1 to 3% after 30 minutes, and successive increases in the percentages of cells with CLD in Stages 2 and 3. Consistent with the concept that the patterns of CLD organization corresponded to consecutive stages of their dispersion, we found that the percentages of cells with CLD in Stages 2 and 3 in isoproterenol-treated cells changed from 60% and 10% respectively after 10 minutes of exposure, to 30% and 70% respectively after 60 minutes of exposure. These observations further suggest that dispersion is a multistep-process involving discrete morphological stages characterized by varying degrees of interaction between individual CLD.

For a second approach, we sought an objective method for defining CLD cluster dispersion that would avoid potential biases associated with subjective morphological analyses. Reasoning that CLD dispersion could be described by an increase in the number of Plin1-positive objects, we used Slidebook software image analysis tools to quantify the number of such objects/cell. Quantitation of CLD dispersion using this approach (Figure 2D), showed that there was a significant (p<0.001), time dependent, increase in the number of Plin1-positive objects/cell following isoproterenol addition. This increase plateaued at levels that were 4 to 10-times the starting values after about 30 minutes. Although this method does not identify specific morphological stages of dispersion, the overall dispersion time course determined by this approach is similar to that determined by morphological analysis.

To further establish the general agreement between the morphological and objects/cell approaches in describing CLD dispersion, both approaches were used to define the time course of CLD dispersion in response to forskolin activation of adenylate cyclase (Figure S5). These data again show progression of CLD from Stage 1 to Stage 2 and then to Stage 3 that correspond temporally to significant (p<0.001) increases in the number of Plin1 objects/cell. We used combinations of these approaches to further characterize the molecular processes that mediate clustering and dispersion of Plin1-CLD.

### Microtubule-dependence of Plin1-coated CLD Dispersion

**Figure 3:**
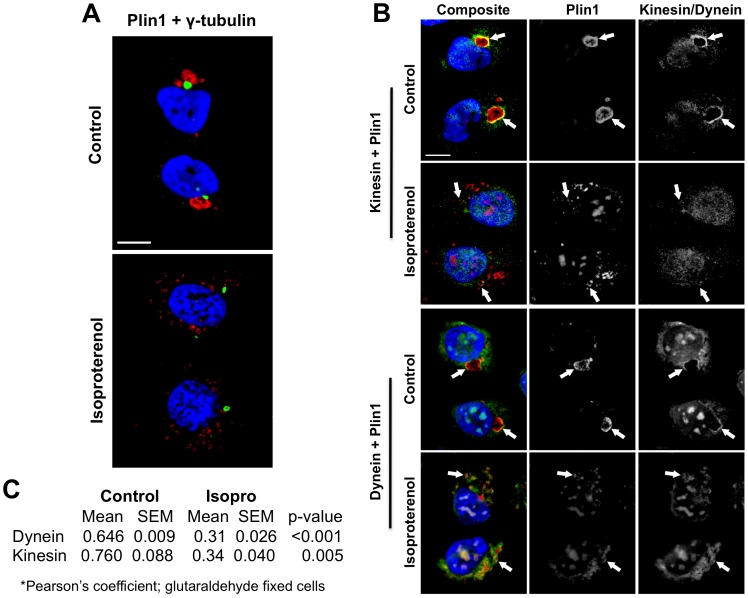
Plin1-coated CLD cluster close to the MTOC and associate with motor proteins. (A) Representative immunofluorescence images showing the localization of Plin1-coated CLD (red) and **γ**-Tubulin (green). Hoechst-stained nuclei are shown in blue. The size bar is 10 µm. (B) Kinesin and dynein co-localization with Plin1. Representative immunofluorescence images depicting the localizations of Plin1 and dynein (Dynein+Plin1) or kinesin-5 family members (Kinesin+Plin1) in Plin1-expressing cells are shown. The panels show merged (red = Plin1; green = dynein or kinesins), and Plin1- and dynein- or kinesin-specific images (monochrome). Arrows point to areas of immunofluorescence overlap seen as a yellow color in the composite. Hoechst-stained nuclei are shown in blue. The size bar is 10 µm. (C) Cross-channel Pearson’s correlation coefficients for the overlap of dynein- or kinesin-immunofluorescence with that of Plin1 in control or isoproterenol-treated (Isopro) cells.

Observations in *Drosophila* embryos and 3T3-L1 cells have documented microtubule dependent CLD movement [24]. In HEK293 cells microtubules emerge from a microtubule organizing center (MTOC) located near the nucleus in interphase cells [25]. The observation that Plin1-coated CLD cluster near the nucleus in unstimulated cells suggested that clustering may occur near the MTOC. We addressed this possibility by immunostaining control Plin1-expressing cells with anti-gamma-tubulin antibody to identify the MTOC [26]. Figure 3A shows that Plin1-coated CLD clusters localize close to foci of gamma-tubulin immunostaining, and therefore appear to be closely associated with the MTOC. In contrast, CLD-clusters did not appear to be associated with markers of the Golgi apparatus, lysosomes, or rough endoplasmic reticulum (Figure S6).

Gamma-tubulin is a microtubule minus-end binding protein [27], therefore clustering of CLD near the MTOC suggests that minus-end directed movement may facilitate this phenomena. Since directed CLD movement in other systems is mediated by the actions of specific plus- or minus-end motors [7], we next investigated motor protein localization relative to Plin1-coated CLD. Figure 3B shows that dynein, a minus-end motor protein, and kinesin-5 family members, which are the plus-end motors found in HEK293 cells, immunolocalize to CLD clusters under control conditions. Dynein and kinesin-5 members also localized to dispersed CLD in isoproterenol-treated cells (Figure 3B). Under both conditions, dynein and kinesin immunostaining exhibit overlap with that of Plin1 suggesting close association. To assess the degree of association, the extent of kinesin and dynein co-localization with Plin1 was quantified by image analysis. Using Pearson’s analysis, a positive correlation was observed for kinesin 5 and dynein immunostaining with Plin1 immunostaining on clustered and dispersed CLD (Figure 3C). The Pearson’s coefficients describing the dynein and Plin1 overlap were similar to those describing the overlap between kinesins and Plin1 on both clustered and dispersed CLD, suggesting that the dispersion and clustering processes are not due to differential association of plus-end and minus-end motors with CLD. However, the degree of overlap for dynein and kinesins with Plin1 was decreased following isoproterenol stimulation, indicating that CLD dispersion was associated with a general decrease in motor proteins association with CLD.

To further investigate the possibility that plus- and minus-end motors co-associate with, and are active, on Plin1-coated CLD we imaged the movement of individual GFP-Plin1-coated CLD in real-time after isoproterenol induced dispersion (Figure S7). The movie shows individual CLD moving very quickly back and forth over long distances without an intervening hesitation before reversal of direction. The lack of a hesitation in the movement suggests that both types of motors are present, and functioning, on individual CLD. In addition, the movement of these CLD appears to traverse the same path when proceeding forward and backward suggesting a constrained path over which they travel, for instance tethering to an intact microtubule network.

**Figure 4:**
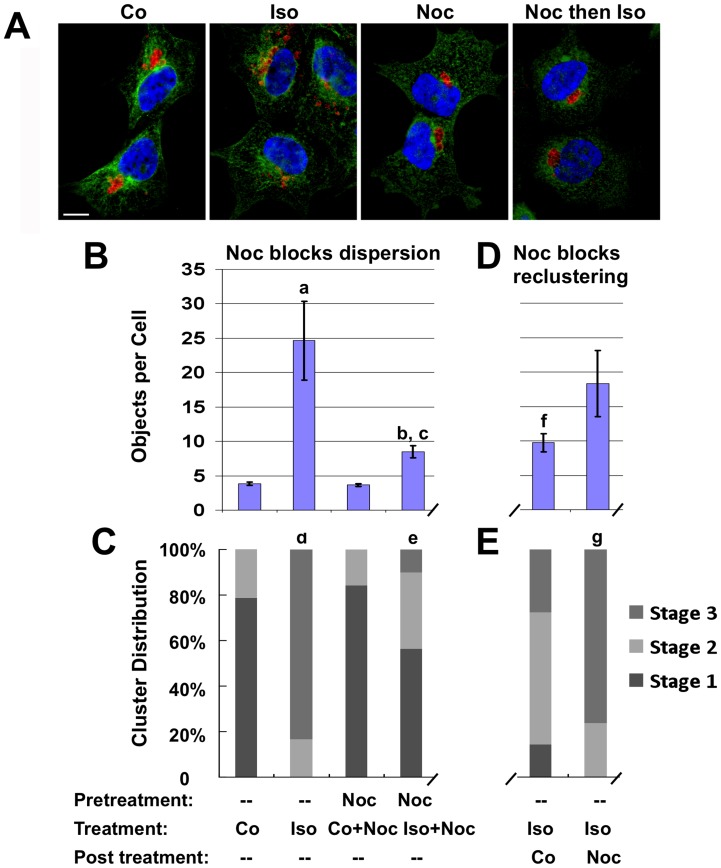
Dispersion and reclustering of the Plin1-coated CLD is inhibited by nocodazole. (A) Representative images of Plin1 (red) and β-tubulin (green) immunofluorescence in Plin1-expressing cells treated with vehicle (Co), 10 µg/ml isoproterenol for 1 hour (Iso), 0.2 µg/ml nocodazole for 20 minutes (Noc), or 0.2 µg/ml nocodazole for 20 minutes then 0.2 µg/ml nocodazole plus 10 µg/ml isoproterenol for 60 minutes (Noc then Iso). Hoechst-stained nuclei are shown in blue. The size bar is 10 µm. (B) Quantification of the effects of nocodazole on isoproterenol stimulated dispersion. The bars show the average number of Plin1 objects/cell (± SEM) for 3 experiments performed in duplicate in cultures that were not pretreated, or that were pretreated with 0.2 µg/ml nocodazole for 20 minutes, and then incubated in control media (Co), media containing 10 µg/ml isoproterenol (Iso), control media containing 0.2 µg/ml nocodazole (Noc+Co), or media containing 0.2 µg/ml nocodazole and 10 µg/ml isoproterenol (Iso+Noc). Treatment conditions for B are shown below C. Statistically significant differences are indicated by the lower case letters a-c: a, control versus isoproterenol-treated (p<0.001); b, isoproterenol-stimulated cultures in the absence versus presence of nocodazole (p<0.01); c, nocodazole-pretreated cultures in the absence versus the presence of isoproterenol (p<0.01). (C) The effects of nocodazole on CLD cluster stages monitored by morphological analysis. The bars show the percentage of CLD in Stages 1–3 for cultures treated as described in panel B. The values are averages of 3 replicate experiments in which 60–80 cells per experiment were analyzed for each condition. Statistical significance is indicated by lower case letters d and e: d, CLD distribution in isoproterenol-treated cultures differs from controls (p<0.001); e, CLD cluster distribution in cells preincubated with nocodazole and treated with nocodazole in the presence of isoproterenol differs from that of cells preincubated with nocodazole and treated with nocodazole in control medium (p<0.001). (D) Quantification of the effects of nocodazole on CLD reclustering. The bars show the average number of Plin1 objects/cell (± SEM) for 3 experiments performed in duplicate in which cultures were treated with isoproterenol for 60 minutes to induce dispersion, washed twice with control medium, and then allowed to recluster in control medium in the absence (Co) or presence of nocodazole (Noc) for 6 hours. The values in D correspond to the y-axis values shown in B. The treatment conditions for D are shown below E. The lower case letter f indicates that the number of Plin1 objects/cell in cultures allowed to recluster in the absence of nocodazole are significantly different from that found for isoproterenol-treated cultures prior to reclustering (p<0.05). The average number of Plin1 objects/cell in cultures that were allowed to recluster in the presence of nocodazole was not significantly different from that of isoproterenol-treated cultures prior to reclustering. (E) The effects of nocodazole on CLD reclustering monitored by morphological analysis. The bars show the percentage of CLD in Stages 1–3 for cultures treated as described in D. The values are averages of 3 replicate experiments in which 60–80 cells per experiment condition were analyzed for each condition. The lower case letter g indicates that the CLD distribution in cells in which CLD reclustered in the presence of nocodazole was significantly different from that of cultures in which reclustering occurred in the absence of nocodazole (p<0.001).

To verify that microtubule-dependent movement mediates cluster dispersion, we next treated cells with nocodazole to disrupt the microtubule network [28]. Figure 4A shows immunohistochemically that incubation with 0.2 µg/ml nocodazole disrupts the microtubule network but does so without affecting the tight CLD-cluster morphology itself. This result suggests that maintenance of CLD as tight clusters in the unstimulated state does not require an intact microtubule network and is not dependent on motor protein actions. However, pre-treating cells with this concentration of nocodazole for 20 minutes prior to adding isoproterenol impaired cluster dispersion. The effects of nocodazole on the dispersion of CLD are quantified in Figure 4B. Under control conditions, there were 3–4 Plin1 objects/cell whether nocodazole was present or not. In cultures stimulated with isoproterenol for 1 hour in the absence of nocodazole, this number rose to approximately 25 objects/cell. In contrast, in cultures pretreated with nocodazole prior to isoproterenol stimulation in the presence of nocodazole, the number of objects/cell averaged about 8, which was significantly fewer (p<0.001) than that found in isoproterenol-treated cultures in the absence of nocodazole. However, the number of objects/cell in cultures pretreated with nocodazole prior to adding isoproterenol was also significantly greater (p<0.01) than that found for control cells, in the absence or presence of nocodazole, which indicates that nocodazole disruption of microtubules does not completely block the effects of isoproterenol on CLD clustering. We used morphological stage analysis to further define the effects microtubule disruption on CLD cluster properties (Figure 4C). Under control conditions, in the presence or absence of nocodazole, about 80% of the cells CLD were in Stage 1 and in the remaining cells they were in Stage 2. In cultures incubated with isoproterenol in the absence of nocodazole for 1 hour, approximately 83% of the cells had completely dispersed Stage 3 CLD, and the remaining cells had CLD in Stage 2. This distribution of CLD differed significantly (p<0.001) from that found for control cultures. In cultures that were pretreated with nocodazole prior to addition of isoproterenol for an hour, we found that approximately 55% of the cells had Stage 1 clusters, 35% had Stage 2 clusters and 10% had fully dispersed CLD. This CLD distribution was significantly different from that found for isoproterenol-treated cultures in the absence of nocodazole (p<0.001) and from untreated cultures (p<0.001). Together, the objects/cell and morphological analyses verify that hormone-induced dispersion of Plin1 CLD in HEK293 cells is microtubule dependent and suggest that additional mechanisms may be involved in the initial declustering step of CLD dispersion.

Since an intact microtubule network is required for isoproterenol-induced cluster dispersion, we next tested whether Plin1 coated CLD recluster, and if an intact microtubule network was also necessary for this process. For these experiments, we treated Plin1-expressing cells with isoproterenol for 1 hour to allow CLD to completely disperse. The cells were then washed to remove the isoproterenol and incubated in fresh control medium without or with 0.2 µg/ml nocodazole for 6 hours. The data in Figure 4D show that in the absence of nocodazole, the number of objects/cell decreased significantly (p<0.05) from approximately 25 to near 10 during the chase period, which is indicative of CLD reclustering. In contrast, when nocodazole was present during the chase period the number of Plin1 objects/cell were not significantly different from that found for fully dispersed CLD at the beginning of the chase period, suggesting that reclustering was impaired or prevented. Consistent with these interpretions, morphological analyses (Figure 4E) revealed that in the absence of nocodazole, the majority of cells possessed clustered CLD following the chase period; with 14% of the cells having Stage 1 clusters, 58% having Stage 2 clusters and 28% have CLD in Stage 3. Whereas in cells that were incubated in media containing 0.2 µg/ml nocodazole during the chase period, we failed to detect Stage 1 clusters in any cells and found that only 24% of the cells possessed Stage 2 clusters, whereas 76% contained Stage 3 CLD. The stage-distribution of CLD reclustered in the presence of nocodazole was significantly different (p<0.001) from that found for cultures reclustered in its absence, but it was not quantitatively different from that found for cultures at the start of the chase period, when CLD were fully dispersed (Figure 4C).

These findings indicate that microtubule disruption interferes with the initial phase of cluster reformation following isoproterenol removal. It was not possible to determine if disrupting the microtubule network completely blocked formation of Stage 1 clusters, or simply slowed the rate of cluster formation, since significant cell loss occurred with exposure to nocodazole for longer than 6 hours. However, during a 16-hour incubation in media lacking nocodazole slightly greater than 50% of the cells reformed Stage 1 clusters. Including 8 µM triacsin C, an inhibitor of triglyceride (TAG) synthesis [21] that blocks CLD formation in HEK293 cells [20], in the chase media did not prevent cluster reformation after 16 hours (data not shown), suggesting that reclustering observed over this period is not dependent on TAG synthesis or *de novo* CLD formation. These data provide evidence that CLD are able to recluster following isoproterenol-induced dispersion, and that reclustering is a microtubule dependent process. Furthermore, the reclustering data suggest that the process of cluster reformation involves at least two kinetically distinct steps; a relatively rapid formation of partially disaggregated clusters and slower formation of tight clusters. Collectively, our data indicate that interconversion between partially declustered (Stage 2) and fully dispersed (Stage 3) CLD is completely dependent on the presence of an intact microtubule network. Although microtubules are also likely to play a role in the transitions between Stages 1 and 2 (see below), the inability of nocodazole to prevent isoproterenol-induced conversion of Stage 1 to 2 suggests that additional mechanisms may contribute to interconversion between these stages.

### Phosphorylation Dependence of CLD Dispersion

**Figure 5:**
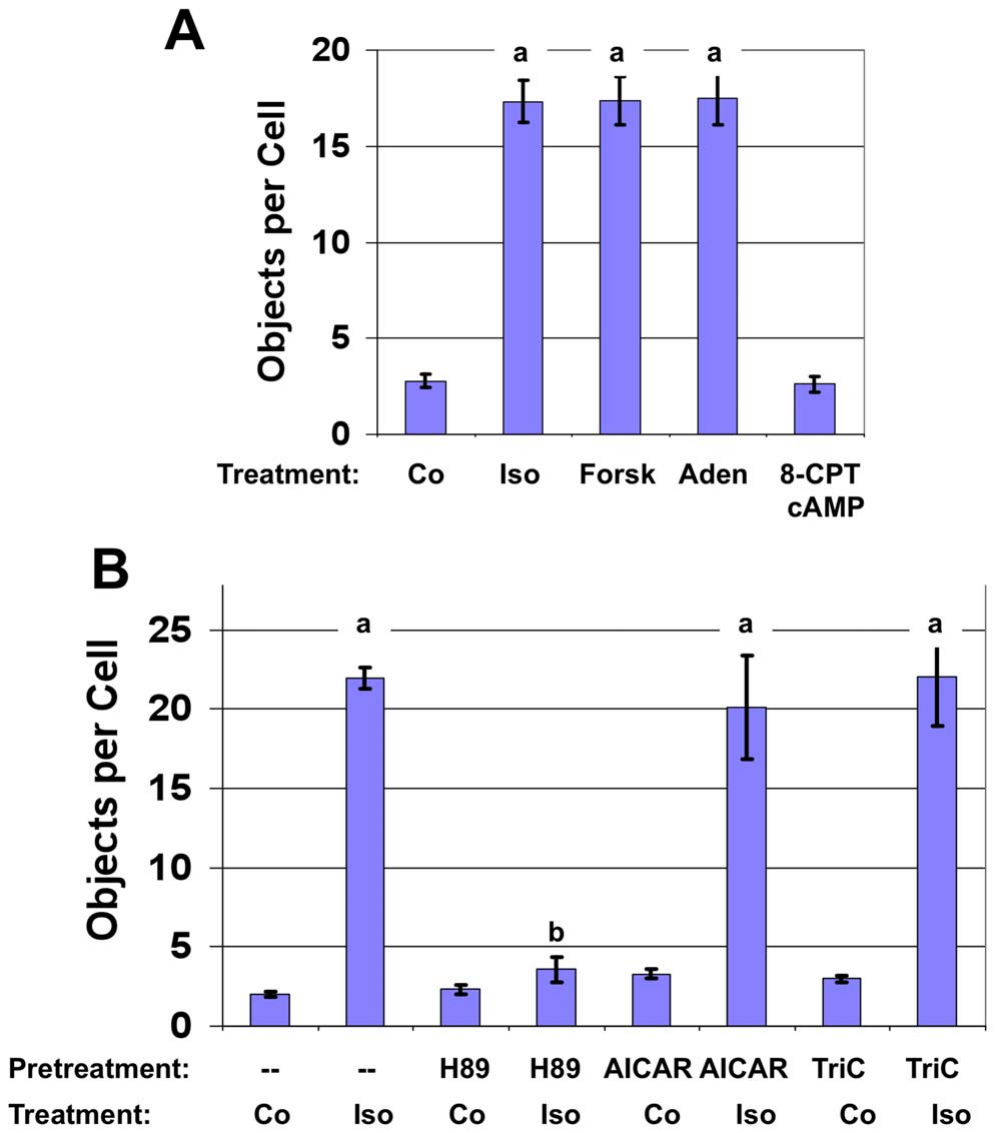
Quantitation of stimulators and inhibitors of Plin1-CLD dispersion. (A) The effects of agents that elevate cAMP on CLD dispersion as determined by the number of Plin1 objects/cell. The values are means ± SEM for 4–10 experiments for Plin1 cultures incubated for 1 hour with: Control media (Co); 10 µg/ml Isoproterenol (Iso); 10 µM Forskolin (Forsk); 10 µM Adenosine (Aden); or 500 µM 8-(4-Chlorophenylthio)-2′-O-methyladenosine-3′,5′-cyclic monophosphate (8-CPT-cAMP). The lower case letter a indicates values that are statistically different from control values, p<0.001. (B) The effects of kinase and triglyceride synthesis inhibitors on CLD dispersion as determined by the number of Plin1 objects/cell. The values are means ± SEM (3 experiments) for Plin1 cultures preincubated without addition (-), or with 20 µM H89, 1 mM AICAR, or 8 µM Triacsin C (TriC) for 30 minutes, and then incubated in control media (Co) or with media containing 10 µg/ml isoproterenol (Iso) for 1 hour. Statistical significance is indicated by lower case letters a and b: a, values differ from their respective controls (p<0.001); b, values for cultures that were preincubated with H89 before being treated with isoproterenol differ from those that were not pretreated before isoproterenol (p<0.001).

Earlier studies suggested that PKA-dependent phosphorylation of Plin1 may initiate the process of CLD dispersion [15]. However, relatively little information exists about the functional relationships between Plin1 phosphorylation and the events involved in CLD dispersion. To better define this relationship, we first compared the effects of various agents that activate adenylate cyclase and stimulate PKA activity on CLD dispersion (Figure 5A). Compared to untreated control cells, isoproterenol, forskolin or adenosine all induced similar degrees of dispersion, presumably through increased intracellular cAMP levels. However, cAMP is capable of eliciting cellular responses through PKA as well as through the guanine exchange factor, Epac [29]. Therefore, we investigated the effects of 8-(4-Chlorophenylthio)-2′-O-methyladenosine-3′,5′-cyclic monophosphate (8CPT-cAMP), a specific stimulator of the Epac system [30], on dispersion. 8CPT-cAMP was not able to stimulate the dispersion process at any concentration up to 500 µM, which is more than 200 fold higher than the EC50 for 8CPT-cAMP activation of Epac1 dose (2.2 µM) [30] (Figure 5A). These results suggest that Plin1-CLD dispersion is specifically linked to cAMP stimulation of PKA dependent phosphorylation.

To define the role of phosphorylation in regulating the specific stages of the dispersion process, Plin1-expressing cells were pretreated with H-89, a selective, cell permeable, competitive inhibitor of protein kinase A activity [31], prior to exposing them to isoproterenol. In control cells, H-89 preincubation for 20 minutes did not affect the number of Plin1 objects/cell indicating that PKA-dependent processes are not involved in maintaining CLD in tight clusters (Figure 5B). In isoproterenol treated cells, H-89 preincubation significantly reduced the number of objects/cell compared to cells that weren’t pretreated with H-89. We also investigated whether other potential regulators of CLD dispersion affected the individual stages of dispersion (Figure 5B). Pre-treating cultures for 30 minutes with aminoimidazole carboxamide ribonucleotide (AICAR), an intermediate in inosine monophosphate generation that acts as an AMP-activated protein kinase agonist [22], did not affect CLD clustering or their isoproterenol-induced dispersion. In addition, we found that pre-treating cultures with triacsin C for 30 minutes did not affect the number of CLD in unstimulated or in isoproterenol-stimulated cultures (Figure 5B), suggesting that short-term exposure to agents that inhibit TAG synthesis also do not affect basal CLD clustering or their ability to be dispersed.

**Figure 6:**
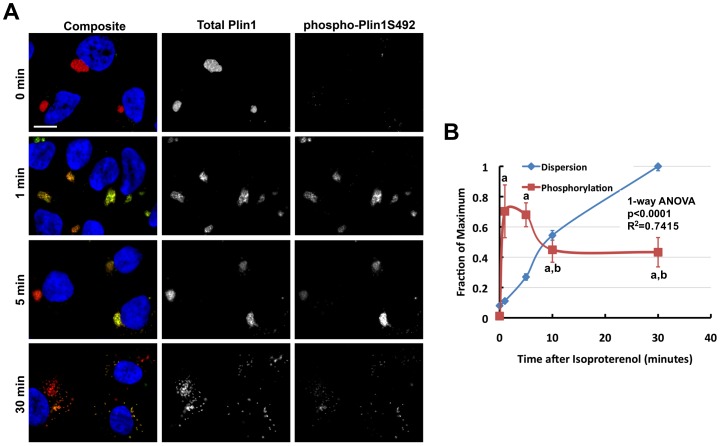
Phosphorylation on Plin1ser492 precedes CLD cluster dispersion. (A) Representative images of Plin1 cells immunostained for Plin1 (red) and phosphorylated Plin1ser492 (phospho-Plin1S492, green) following stimulation for the indicated times with 10 µg/ml isoproterenol. Hoechst-stained nuclei are shown in blue. The size bar is 10 µm. (B) The time courses of CLD dispersion (blue line) and phosphorylation of Plin1S492 (red line). CLD dispersion is shown as the fraction of the total number of Plin1 objects/cell observed at 45 minutes. The extent of Plin1S492 phosphorylation is shown as a relative ratio of phospho-Plin1ser492 fluorescence to total Plin1 fluorescence. The values are mean ± SEM for 6 experiments with the evaluation of 60–100 cells per time point in each experiment. Statistical significances of Plin1 phosphorylation are indicated by lower case letters a and b: a, values differ from the 0 minute value (p<0.001); b, values differ from 0 minutes value (p<0.01) but not from 1 or 5 minute time point values. A 1-way ANOVA of Plin1 objects/cell yields a p<0.0001 and R^2^ = 0.7507, a post test for linear trends was statistically positive with p = 0.0207.

Having established that Plin1-CLD dispersion is induced by adenylate cyclase activation, and is dependent on PKA activity, we next wanted to investigate the role of Plin1 phosphorylation in initiating the dispersion process. Although phosphorylation of Plin1S492 is known to be required for dispersion of clustered CLD in 3T3L1 fibroblasts (15), the quantitative and temporal relationships between phosphorylation at this site and dispersion have not been defined. Accordingly, we examined the time courses of dispersion and the degree of Plin1S492 phosphorylation on individual CLD within single cells. Figure 6A shows representative images of Plin1 cells immunostained with antibodies to Plin1 and phospho-Plin1S492 following stimulation with isoproterenol for varying lengths of time. Prior to isoproterenol addition (0 minutes), Plin1-positive CLD are tightly clustered and lack significant phospho-Plin1S492 staining. Within 1 minute after isoproterenol addition, we found the majority of CLD were positive for phospho-Plin1S492. At this time virtually all of the CLD remained as tight clusters. By 5 minutes, the majority of CLD continued to remain positive for phospho-Plin1S492 and there was now evidence of CLD declustering. After 30 minutes, the clusters were fully dispersed, and at this time many CLD appeared to lack phospho-Plin1S492 staining.

To quantify the temporal relationship between phospho-Plin1S492 and cluster dispersion we determined relative levels of phospho-Plin1S492 (phospho-Plin1S492/Plin1) and the relative degree of cluster dispersion at several time points after isoproterenol exposure (Figure 6B). These data show that the majority (70%) of Plin1 is phosphorylated on S492 after 1 minute of isoproterenol exposure, and that it remains highly phosphorylated at this site for up to 5 minutes after exposure. The extent of CLD dispersion, as measured by the number of Plin1-positive objects/cell, exhibited a significant linear trend throughout the experiment, however it increased only a very small amount in the first minute whereas the phosphorylation of Plin1S492 changed from near zero to its maximum during this time. Between 5 and 10 minutes after isoproterenol addition, the fraction of phospho-Plin1S492 decreased to about 40% and remained at this level to 30 minutes. During this time, CLD dispersion continued to increase, reaching its maximum at approximately 30 minutes. These results demonstrate that in response to isoproterenol stimulation the majority of Plin1 undergoes rapid S492 phosphorylation before significant changes occur in CLD clustering. The relationship between the extent of Plin1 phosphorylation on S492 and the degree of dispersion was further examined at the level of individual cells at the same time points after isoproterenol addition (Figure S8). These data show that at initial time points following isoproterenol addition the vast majority of cells possess phospho-Plin1S492, but that at later stages of dispersion that there is wide variation in the degree of Plin1S492 phosphorylation and that many cells have dispersed CLD now coated by Plin1 that is no longer phosphorylated on S492.

The time course data in Figure 6B also indicate that the process of CLD declustering and dispersion is initiated between 1–5 minutes after isoproterenol addition. To define the initial steps of this process in greater detail, we performed 4D imaging of declustering responses of GFP-Plin1 clusters in real-time, at 1 image/sec over the course of the first 8 minutes after isoproterenol stimulation (Figure S9). The movie shows the temporal response of a representative GFP-Plin1 CLD cluster to isoproterenol addition. The main cluster remains virtually intact for 2.5 minutes and then begins to perceptibly loosen, after which time, individual CLD and smaller CLD clusters can be seen to break-off and move away from the main cluster. Individual CLD can also be seen to move away from, and then reform with, the main clusters and with smaller clusters, suggesting that even at the earliest stages of dispersion, CLD undergo rapid anterograde and retrograde movement. Combined with time course analysis of fixed cells (Figure 6B), these real-time data support the concept that phosphorylation of Plin1 precedes the earliest changes in cluster morphology associated with their dispersion.

### Moesin Regulation of CLD Clustering

**Figure 7:**
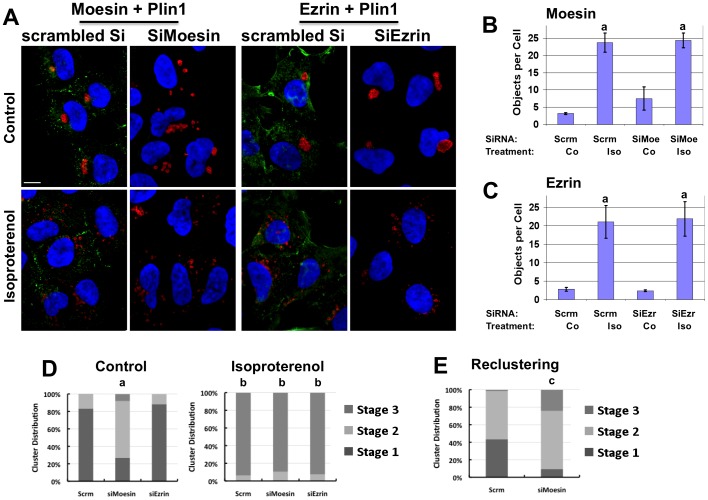
Moesin is required for Plin1-CLD clustering. (A) Immunolocalization of Plin1CLD (red) and moesin or ezrin (green) in cells transfected with scrambled oligonucleotides (scrambled Si) or siRNA oligonucleotides to moesin (SiMoesin) or ezrin (SiEzrin) and exposed to vehicle (Control) or 10 µg/mL isoproterenol for 1 hour (Isoproterenol). Hoechst-stained nuclei are shown in blue. The size bar is 10 µm. (B and C) CLD dispersion as determined by the number of Plin1 objects/cell in cultures transfected with scrambled siRNA (Scrm) or with siMoesin (B, SiMoe) or siEzrin (C, SiEzr) oligonucleotides for 48 hours and then incubated for 30 minutes with control medium (Co) or medium containing 10 µg/mL isoproterenol isoproterenol (Iso). The values are mean ± SEM for 3 experiments performed in duplicate. The lower case letter a above the bars indicates values that differ from their respective controls (p<0.001). (D) The effects of moesin or ezrin knockdown on CLD distribution determined by morphological analysis. The average CLD distribution among Stages 1–3 is shown for cultures transfected with scrambled (Scrm), siMoesin and siEzrin oligonucleotides for 48 hours and then exposed to vehicle (Control) or isoproterenol (Isoproterenol) as described above in B and C. The values are averages of 3 replicate experiments in which 60–80 cells per condition were analyzed per experiment for each condition. Statistical significance is indicated by lower case letters a and b: a, CLD distribution in vehicle-treated cultures transfected with siMoesin oligonucleotides was significantly different (p<0.001) from that found in cultures transfected with scrambled or siEzrin oligonucleotides; b, CLD distributions in isoproterenol treated cultures were significantly different from that found in the respective control cultures (p<0.001). (E) The effects of moesin knockdown on CLD reclustering. The average CLD distribution among Stages 1–3 is shown for cultures transfected with scrambled (Scrm) or siMoesin oligonucleotides (siMoesin) following isoproterenol dispersion for one hour, washing twice, and then incubation in control medium for 16 hours to allow reclustering. The values are averages of 3 replicate experiments in which 60–80 cells per condition were analyzed per experiment for each condition. The lower case letter c indicates CLD distributions in scrambled oligonucleotide transfected cultures that are significantly different (p<0.001) from those transfected with siMoesin oligonucleotides.

Movement of cellular organelles through the cytoplasm is hypothesized to switch back and forth between microtubules and actin filaments [32]. Ezrin and moesin, members of the ezrin-radixin-moesin (ERM) family of actin-associated proteins, are implicated in the control of cortical actin functions [33], and both proteins are reported to be important for stable microtubule formation [34], [35]. Therefore, we sought to determine if ERM proteins play a role in Plin1-CLD clustering and/or dispersion by blocking expression of ezrin or moesin using validated siRNA oligonucleotides. In Plin1 cells transfected with scrambled oligonucleotides, ezrin and moesin were immunolocalized near the plasma membrane and within the cytoplasm as expected (Figure 7A, green fluorescence). Transfection with scrambled oligonucleotides did not affect CLD clustering or interfere with isoproterenol-stimulated CLD dispersion. Forty-eight hours following transfection with their respective siRNA oligonucleotides, we were unable to detect ezrin or moesin by immunostaining (Figure 7A), suggesting that their levels were significantly depleted by this time. Inhibiting ezrin expression did not affect CLD clustering or interfere with CLD dispersion following isoproterenol stimulation (Figure 7C). By contrast, under control conditions, inhibiting moesin expression disrupted the tightly clustered CLD morphology producing significant shifts in the number of cells with multiple small-cluster (Stage2) CLD morphology (Figure 7A). The effects of moesin knockdown on cluster dispersion properties were quantified using both morphological and Plin1 objects/cell approaches. In the absence of isoproterenol, the number of Plin1 objects/cell increased from about 2.5 in cultures transfected with scrambled siRNA to around 8 in cultures transfected with moesin siRNA (Figure 7B), which is consistent with partial disruption of Stage 1 clusters. Using the morphological assay, we found a significant increase (p<0.001) in the fraction of CLD in Stages 2 and 3 in moesin siRNA-transfected cultures compared to scrambled siRNA transfected cultures under control conditions (Figure 7D). However, moesin knockdown did not significantly affect the number of Plin1 objects/cell (Figure 7B), or alter formation of Stage 3 CLD in cells treated with isoproterenol (Figure 7D).

**Figure 8:**
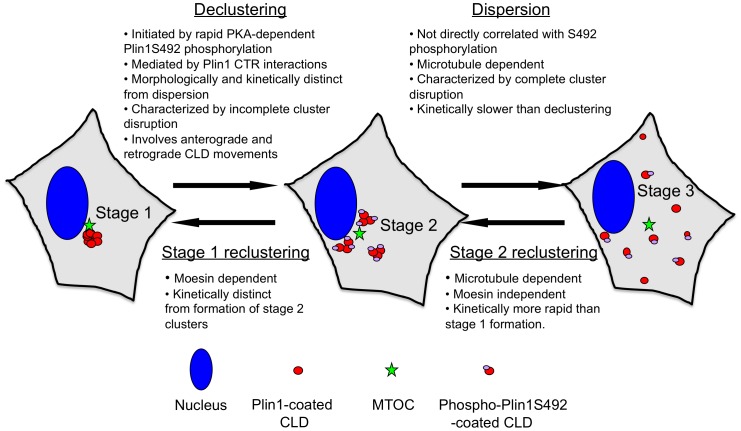
CLD clustering and dispersion mechanisms. Tight clusters of Plin1-coated CLD (red spheres) form near the MTOC (green stars) by moesin- and microtubule dependent processes in unstimulated cells (Stage 1). PKA activation induces rapid phosphorylation (less than a minute) of Plin1S492 (small purple ovals on red spheres), initiating processes that lead to disruption of CLD interactions and their separation into smaller clusters over a 4–10 minute period (Stage 2). (Stage 3), Mictroubule-movement of fully- or partially-disaggregated CLD leads to their dispersion over the course of 20–40 minutes. CLD movement on microtubules is not dependent on phosphorylation of Plin1 on S492 (indicated by red spheres with out purple ovals in the Stage 3 cell). Dispersed CLD undergo reclustering to Stage 2 and Stage 1 by kinetically distinct mictrotubule- and moesin-dependent processes.

These results indicate that moesin contributes to formation of CLD clusters but it does not appear to be involved in their dispersion. To further evaluate this possibility, we examined the effects of moesin knockdown on reformation of Stage 1 clusters from fully dispersed CLD after isoproterenol removal. Plin1 cells were transfected with scrambled oligonucleotides or siRNA moesin oligonucleotides for 48 hours and stimulated with isoproterenol for 60 minutes to induce complete dispersion of CLD. Isoproterenol was then washed out, and the cultures were treated with control medium for 16 hours. Figure 7E shows that at this time about 45% of the cells transfected with scrambled siRNA had CLD in Stage 1, and that in the remaining cells CLD were in Stage 2. In contrast, in cells transfected with siRNA to moesin, about 10% of the cells contained Stage 1 CLD, 66% had CLD in Stage 2, and 24% remained fully dispersed (Stage 3). Thus reformation of Stage 1 but not Stage 2 clusters appears to depend, in part, on moesin expression. Collectively the data in Figure 7 provide evidence that, in addition to the Plin1 CTR, moesin may contribute to CLD cluster formation.

## Discussion

Plin1 regulates basal and hormone-stimulated lipolysis in adipose tissue through phosphorylation dependent mechanisms [11]. Emerging evidence suggests that hormone-stimulated lipolysis involves reorganization and dispersion of lipid droplets [15], [36], [37] and that Plin1 phosphorylation may control these processes [15]. In this study, we investigated the dynamics and molecular processes governing β-adrenerigic-stimulated dispersion of Plin1 coated CLD in cell culture models. Our data document that Plin1 governs clustering and hormone-stimulated dispersion of CLD in HEK293 cells, and that these actions map to the carboxy-terminal region of Plin1 and are regulated by PKA-dependent phosphorylation. We further demonstrate that the dynamics of CLD dispersion are complex, involving multiple-steps that include; rapid phosphorylation of Plin1 on S492, alterations in CLD cluster organization, and activation of both anterograde and retrograde movement of individual and clustered CLD. Based on these findings, and evidence that Plin1S492 phosphorylation is specifically required for dispersion of clustered CLD in 3T3-L1 fibroblasts [15], we propose a sequential model of hormone-stimulated CLD dispersion that involves at least three temporally distinct processes: (1) rapid phosphorylation of Plin1S492; (2) disruption of interactions that mediate CLD clustering; and (3) disaggregation and dispersion of CLD by microtubule-dependent movement.

### The Plin1 CTR Controls Clustering and Hormone-stimulated Dispersion of CLD

Residues within the Plin1 CTR were proposed to mediate interactions that induce CLD clustering in 3T3-L1 fibroblasts [16]. This concept is supported by our data showing CLD coated with Plin1(198–517)-VSV form tight clusters in control HEK293 cells, whereas CLD coated by Plin1(1–197)-VSV are fully dispersed. Further, in conjunction with observations that phosphorylation of Plin1S492 is required for isoproterenol- or forskolin-induced dispersion of Plin1-coated CLD [15], our data provide evidence that the Plin1-CTR interactions that mediate CLD clustering are somehow disrupted following Plin1S492 phosphorylation. Thus, in contrast to triglyceride storage and lipolysis regulatory functions of Plin1, which involve both N- and C-terminal regions of Plin1 [16], [23], the CLD clustering and dispersion functions of Plin1 appear to specifically localize to residues, or sequences, within its C-terminal region. Interestingly, the protective functions of Plin1 against lipolysis have also been mapped to its CTR [23], [38]. Further molecular dissection of this region by mutagenesis will be necessary to determine if these functions are mediated by the motifs involved in mediating clustering and hormone-dependent dispersion of CLD.

### Plin1S492 Phosphorylation Controls CLD Dispersion by Disrupting Clustering Interactions

The nature of the Plin1-CTR interactions mediating CLD clustering, and how S492 phosphorylation, and possibly phosphorylation of other Plin1 sites [39], induces cluster dispersion, remain open questions. It has been suggested that Plin1 forms a dynamic scaffolding around CLD that controls the access of enzymes and adaptor proteins to the CLD surface in response to changes in its phosphorylation state [11]. Phosphorylation-dependent alterations in Plin1 scaffolding properties could induce CLD dispersion by disrupting homotypic Plin1 CTR interactions that mediate clustering, thereby permitting CLD movement on attached microtubules. Alternatively, Plin1 phosphorylation could mediate dispersion by inducing CLD attachment to microtubules, either directly or through the actions of adaptor proteins. Our data provide evidence that helps to distinguish between these possibilities. First, our observations that in unstimulated cells, CLD clusters localize near the MTOC, and that both kinesin- and dynein-motors are associated with CLD in their clustered state, suggest that Plin1 phosphorylation is not required to induce coupling of CLD to microtubule transport machinery. Second, our time course studies show that there is a significant time lag between isoproterenol stimulated Plin1S492 phosphorylation and evidence of CLD movement or cluster dispersion, demonstrating that phosphorylation at this site is not associated with immediate changes in CLD motility that lead to cluster disaggregation and/or dispersion. Third, we found that during the period of maximum CLD dispersion that Plin1 phosphorylation on S492 did not directly correlate with the degree of dispersion, suggesting that once dispersion was induced, S492 phosphorylation was no longer be required. Fourth, 4D imaging studies of the initial phase of cluster dispersion showed that clusters visibly loosen and undergo morphological changes prior to evidence of directed CLD movement or dispersion. Collectively these data support a model of hormone-stimulated CLD dispersion, in which Plin1S492 phosphorylation plays a permissive role in regulating dispersion by disrupting interactions that mediate CLD clustering.

Plin1S492 undergoes nearly complete phosphorylation within the first minute after isoproterenol stimulation, suggesting a highly efficient process that is tightly coupled to β-adrenergic activation in Plin1 expressing cells. The rapidity of this phosphorylation compared with the time courses of CLD declustering or dispersion further indicates that it may initiate these processes. Although additional studies are required to establish the mechanism of this initiation, the lag between Plin1S492 phosphorylation and the first evidence of cluster movement suggest that phosphorylation *per se* does not directly induce declustering or dispersion, and that it is not rate-limiting for these processes. Plin1 phosphorylation has been shown previously to induce changes in CLD coat-protein properties related to increased lipolytic activity [5], including translocation of HSL to the CLD surface [40], [41], and disruption of Plin1-CGI58 interactions [41]. The timing of these changes, which appear to be maximal within 4–10 minutes after stimulation [41], correspond with that of the initial declustering phase of isoproterenol-induced CLD dispersion observed in our study. Whether such changes specifically contribute to cluster dispersion, or are simply coincidental with it, remains to be determined. However, isoproterenol stimulation is known to induce significant changes in the protein composition of CLD in adipocytes [5]. Thus there is significant circumstantial evidence supporting a mechanism in which modification of CLD coat-protein properties by Plin1 phosphorylation disrupts interactions between CLD that lead to their declustering and subsequent microtubule-dependent movement. Our observations that some CLD remain clustered during movement, and remain clustered for extended periods of time after isoproterenol stimulation, indicate that the processes mediating declustering are not as efficient as those responsible for Plin1S492 phosphorylation, and demonstrate that complete declustering is not required for microtubule-dependent movement of CLD.

### The Role of Moesin and Microfilaments in CLD Clustering and Dispersion

The actin cytoskeleton and microtubule networks are known to cooperate in regulating the transport and specifying the cellular locations of cargo [32], [42]. Our observation that knockdown of moesin induces disaggregation of large CLD clusters into smaller ones suggests that the microfilament network may contribute to formation of tight CLD clusters. How moesin influences CLD clustering is still unclear, however evidence from the literature suggest two possible mechanisms for its function. First, moesin may contribute to cluster stabilization through effects on actin filaments. Disruption of this filament organization may therefore be an initial step in their dispersion. Actin-microtubule cross talk, involving both structural and regulatory interactions, is known to contribute to a number of dynamic cellular processes [32]. Since cAMP is known to promote microtubule assembly and actin microfilament reorganization [43] through kinase independent actions, it is possible that elevated cAMP initiates cluster fragmentation through kinase- dependent and/or –independent, effects on microfilament-microtubule organization.

Secondly, moesin has been postulated to function as a protein kinase A anchoring protein (AKAP) [44]. AKAP proteins, which regulate spatial and temporal control of PKA activation by targeting pools of PKA to distinct subcellular locations [45], are known to enhance β-adrenergic activation of PKA [46]. Although further work will be required to determine if moesin’s AKAP functions contribute to the efficiency by which Plin1-S492 is phosphorylated following isoproterenol stimulation, this possibility is supported by moesin localization to the plasma membrane, the rapid phosphorylation of Plin1-S492, and by recent evidence showing that the AKAP protein, optic atrophy 1, organizes a supramolecular complex containing PKA and Plin1 in 3T3L1 cells [47].

### Microtubule Dependence of CLD Declustering and Dispersion

In contrast to the random oscillating movements of individual CLD observed in most cultured mammalian cells [7], the dispersion of Plin1-coated CLD in both 3T3L1 and HEK293 cells appears to occur by processes that are coordinated and directional. Previous models of CLD movement have envisioned individual CLD moving along microtubule tracks [7]. Coupled with data demonstrating that nocodazole disruption of microtubules inhibits CLD dispersion, our live cell imaging results showing that large CLD clusters break apart into smaller clusters that move apart from one another, provide the first evidence that clustered CLD also move along microtubule tracks.

Differential association and differential activation of plus- or minus-end directed motors have been proposed as alternative mechanisms to explain directed microtubule movement of CLD, and other cargo [17]. In our system, directed CLD dispersion does not appear to be associated with differential microtubule motor protein recruitment to these structures. We found similar amounts of plus- and minus-end motors on clustered CLD; and although their association with CLD appeared to decrease following dispersion, the extent of decrease was similar for both types of motors. Coupled with real time imaging results showing that both individual and clustered CLD rapidly switch between anterograde and retrograde movement, these data provide evidence that CLD movement is regulated by alternating activation of plus- and minus-end directed motors. Furthermore, the observations that CLD exhibit anterograde and retrograde movement during the initial phase of declustering, as well as after they are fully dispersed, demonstrate that switching between plus- and minus-end directed movement occurs throughout the dispersion process. How activation of plus- and minus-end directed motors is regulated to achieve directed CLD movement during dispersion is unknown. Further work is necessary to determine whether elevated cAMP levels influence the direction of CLD movement as well as activating the processes that induce their declustering. Curiously, the directional movement of Weibel-Palade bodies in endothelial cells has also been shown to be cAMP regulated [48].

Work by others utilizing *Drosophila* embryos suggests that the Plin1 homologue LSD-2 directly interacts with plus- and minus-end motor proteins to regulate CLD transport [17]. Consistent with this concept, we found that plus- and minus-end motor protein immunostaining overlapped with that of Plin1 on both clustered and dispersed CLD. We also found that CLD coated with Plin2 or Plin3, and that lack Plin1, undergo retrograde- and anterograde-directed movement (data not shown). Additional work will be required to determine whether motor proteins directly interact with mammalian PLIN family members; and if this is the case, if such interactions involve common interaction motifs.

In summary, we show that the CTR of Plin1 mediates CLD clustering and dispersion in HEK293 cells, and that dispersion involves multiple, temporally distinct steps, that are induced by adenylate cyclase activation and dependent on PKA activity. We further identify a role for moesin in stabilizing clusters, and demonstrate that cluster dispersion is complex involving coordinated plus- and minus-end directed microtubule-dependent movement. Additional work is needed to validate the individual roles of these processes in adipocytes and intact adipose tissue, and to understand how dispersion and clustering of individual CLD are integrated with the processes controlling breakdown and reformation larger fat droplets during lipolysis-lipogenesis cycles [49]. Nevertheless, the stages of isoproterenol-induced CLD dispersion in HEK293 cells appear to be similar to those described for forskolin-induced dispersion in 3T3-L1 fibroblasts [15], suggesting that many of the fundamental processes governing CLD clustering and dispersion may involve common underlying mechanisms. Our data provide additional evidence of the unique role played by Plin1 in regulating CLD clustering, as well as new insight into the cellular machinery and processes that determine CLD trafficking patterns during PKA-induced dispersion.

## Supporting Information

Figure S1 **Immunoblot of Plin1 following 10 minutes of isoproterenol stimulation.** Immunoblots of Plin1 and β-actin in extracts of Plin1 cells incubated with 10 µg/ml isoproterenol (Iso), or vehicle (Co) for 10 minutes then harvested and prepared for immunoblot analysis. The lower arrow indicates the migration position of non-phosphorylated Plin1; the upper arrow indicates the up-shifted migration position of phosphorylated Plin1.
(TIF)

Figure S2 **Plin1-coated CLD dispersion does not require Plin2**. Representative immunofluorescence images of CLD localization in Plin1+Plin2 expressing cells cultured for 48 hours in control media (CM) or control media supplemented with 100 µM OA (CM+OA) before being exposed to media without (Control) or with 10 µg/ml isoproterenol for 1 hour (Isoproterenol). Plin1 and Plin2-VSV were detected by immunostaining with antibodies to Plin1 (red) and VSV (green) respectively. The panels show merged, Plin1- and VSV-specific images. The arrows indicate the locations of selected CLD clusters or individual CLD that stained for both Plin1 and Plin2-VSV. Hoechst-stained nuclei are shown in blue. The size bar is 10 µm.
(TIF)

Figure S3 **Plin1-coated CLD dispersion does not require Plin3**. Representative images of CLD localization in Plin1-expressing cells that were transfected with scrambled (Scrambled) or Plin3 siRNA oligonucleotides (siPlin3) and cultured in CM or CM+OA. Images are shown for cells before (Control) and after exposure to 10 µg/ml isoproterenol for 1 hr (Isoproterenol). Cells were immunostained for Plin1 (red) or endogenous Plin3 (green). Hoechst-stained nuclei are shown in blue. The size bar is 10 µm.
(TIF)

Figure S4 **Real-Time movie projection of the dispersion of Plin1 coated CLD.** The tile images showing the dispersion of Plin1-coated CLD following Isoproterenol stimulation (Figure 2A) are included here as a real-time movie.
(MOV)

Figure S5 **Forskolin stimulated cluster dispersion.** (A) The change in CLD clustering as a function of time after exposure to 10 µM forskolin monitored by morphological analysis. The values are averages of 3 experiments, in each experiment 60–100 cells were assayed per time point. Statistical significance is indicated by lower case letters a and b above the bars: a, stage values are different from values at previous time points (p<0.001); b, values are different from 0, 2.5, 5, and 10 minute values (p<0.001). (B) CLD dispersion is determined as a function of time after exposure to 10 µM forskolin by quantifying Plin1 objects/cell. Values are means ± SEM for 3 experiments again examining 60–100 cells per time point in each experiment. A 1-way ANOVA analysis of Plin1 objects/cell yields a p<0.0001; the post test for linear trends p<0.0001, and R^2^ = 0.0.8012
(TIF)

Figure S6 **Plin1-coated clustered CLD do not localize near Golgi, lysosomes, or endoplasmic reticular structures.** Representative immunofluorescence images of Plin1 (red) and: Golgi membrane protein 130 (GM130, green); lysosomal membrane associated protein 2 (LAMP2, green); the endoplasmic reticulum protein calreticulin (Calretic, green) in Plin1 cells treated with vehicle (Control) or 10 µg/ml isoproterenol for 1 hour (Isoproterenol). Hoechst-stained nuclei are shown in blue. The size bar is 10 µm.
(TIF)

Figure S7 **GFP-Plin1 coated CLD move back and forth on microtubules.** A representative GFP-Plin1 expressing cell is viewed in real time approximately 30 minutes after Isoproterenol stimulation. The movie shows examples of CLD that move back and forth without apparent hesitation at the end of each directional movement. The size bar is 2 µm.
(M4V)

Figure S8 **Time course single cell analysis of Plin1-S492 phosphorylation following isoproterenol stimulation.** The data show the results of single cell analyses comparing the relative amounts of Plin1 phosphorylated on S492 and the extent of CLD dispersion, as determined from the number Plin1 objects/cell, at time points 0, 1, 5, 10, and 30 minutes. Approximately 50–75 cells per time point are shown. The results from individual cells are represented by a blue diamond, cell averages are represented by red squares. Also shown are graphs for the mean fraction Plin1-S492-phosphorylated and mean dispersion at these same time points following isoproterenol induced dispersion.
(TIF)

Figure S9 **Real time movie for the dispersion process from 0–8 minutes post isoproterenol treatment.** Isoproterenol-induced CLD declustering is shown in a representative GFP-Plin1 expressing cell at 1 frame per second for the first 8 minutes. Temporary movement of the cell out of the focal plane at the beginning of the movie indicates isoproterenol addition.
(M4V)
