# Supplementary figures and images for: Dynamics and Molecular Determinants of Cytoplasmic Lipid Droplet Clustering and Dispersion
Source: PLoS One. 2013 Jun 25;8(6):e66837. doi: 10.1371/journal.pone.0066837 (PMC3692517; doi:10.1371/journal.pone.0066837)

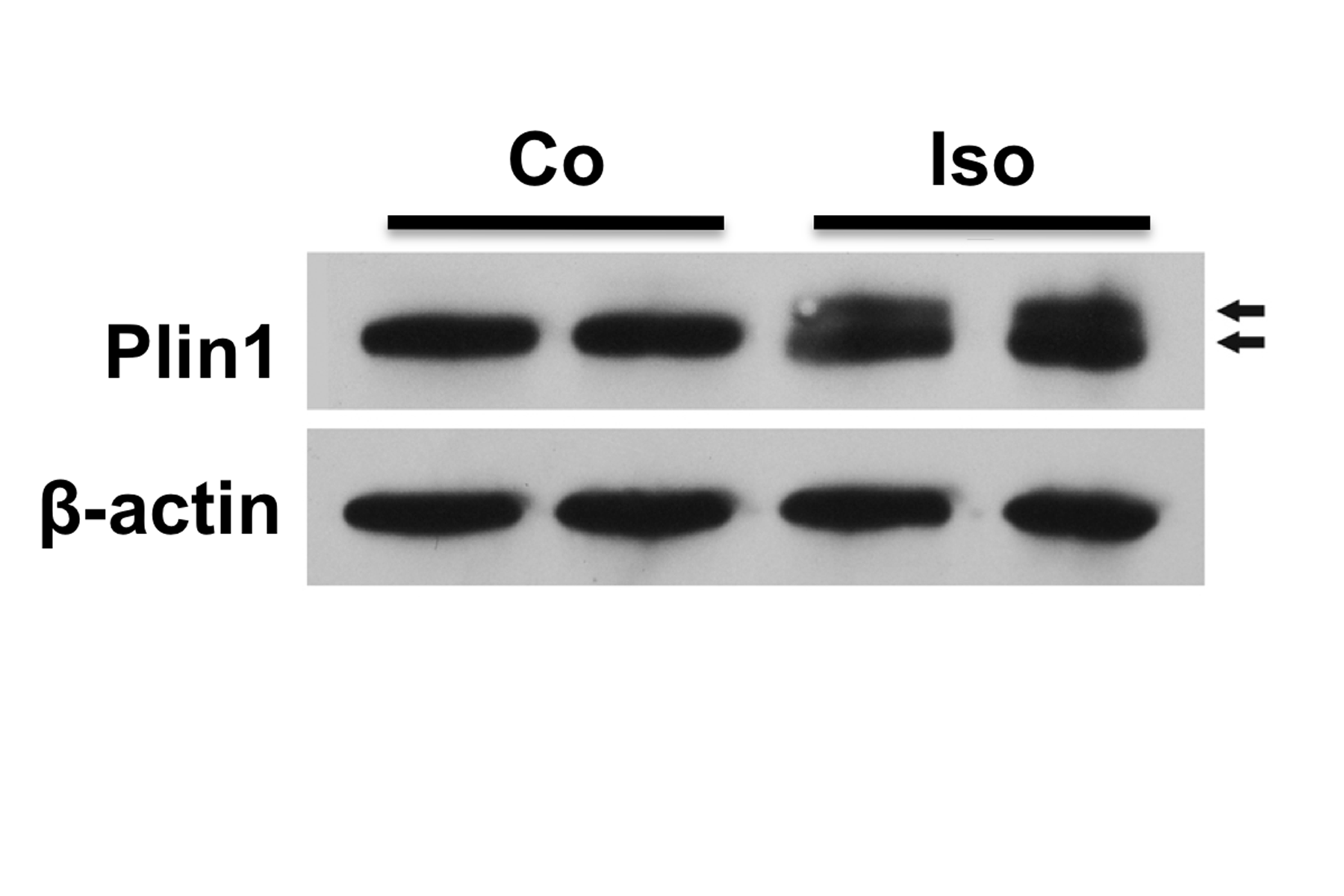

Supplement: Figure S1 — Immunoblot of Plin1 following 10 minutes of isoproterenol stimulation. Immunoblots of Plin1 and β-actin in extracts of Plin1 cells incubated with 10 µg/ml isoproterenol (Iso), or vehicle (Co) for 10 minutes then harvested and prepared for immunoblot analysis. The lower arrow indicates the migration position of non-phosphorylated Plin1; the upper arrow indicates the up-shifted migration position of phosphorylated Plin1. (TIF) [file pone.0066837.s001.tif]

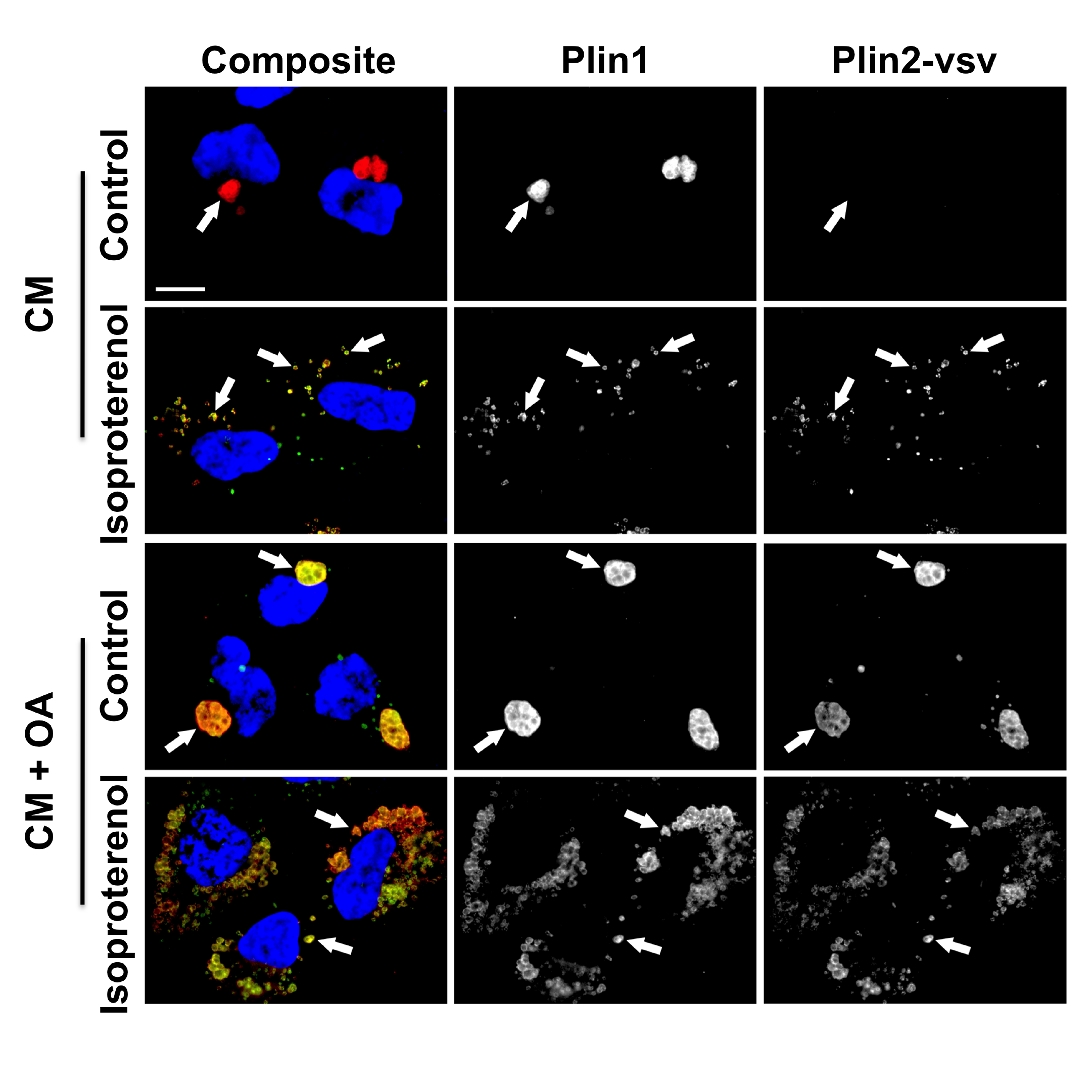

Supplement: Figure S2 — Plin1-coated CLD dispersion does not require Plin2. Representative immunofluorescence images of CLD localization in Plin1+Plin2 expressing cells cultured for 48 hours in control media (CM) or control media supplemented with 100 µM OA (CM+OA) before being exposed to media without (Control) or with 10 µg/ml isoproterenol for 1 hour (Isoproterenol). Plin1 and Plin2-VSV were detected by immunostaining with antibodies to Plin1 (red) and VSV (green) respectively. The panels show merged, Plin1- and VSV-specific images. The arrows indicate the locations of selected CLD clusters or individual CLD that stained for both Plin1 and Plin2-VSV. Hoechst-stained nuclei are shown in blue. The size bar is 10 µm. (TIF) [file pone.0066837.s002.tif]

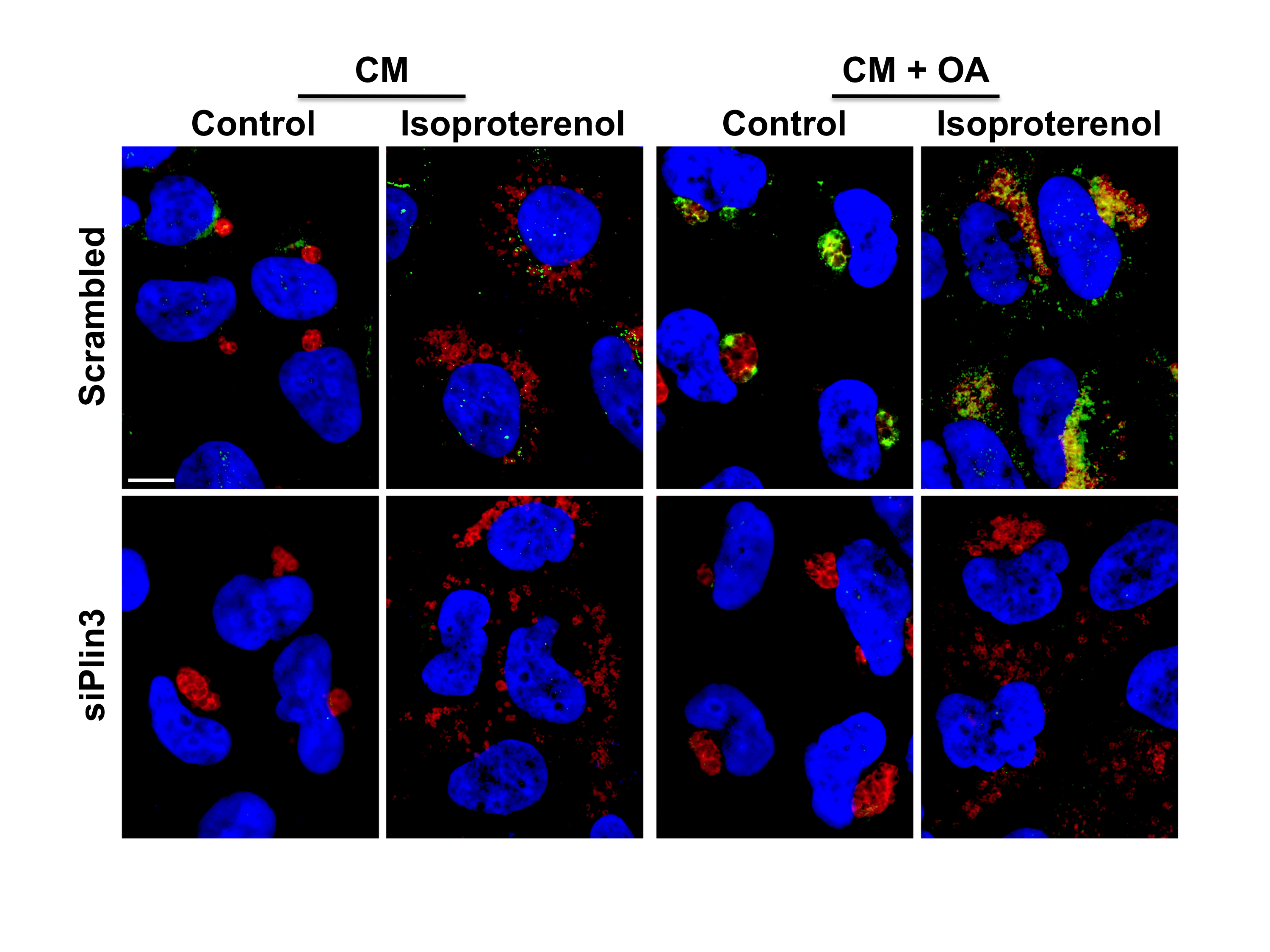

Supplement: Figure S3 — Plin1-coated CLD dispersion does not require Plin3. Representative images of CLD localization in Plin1-expressing cells that were transfected with scrambled (Scrambled) or Plin3 siRNA oligonucleotides (siPlin3) and cultured in CM or CM+OA. Images are shown for cells before (Control) and after exposure to 10 µg/ml isoproterenol for 1 hr (Isoproterenol). Cells were immunostained for Plin1 (red) or endogenous Plin3 (green). Hoechst-stained nuclei are shown in blue. The size bar is 10 µm. (TIF) [file pone.0066837.s003.tif]

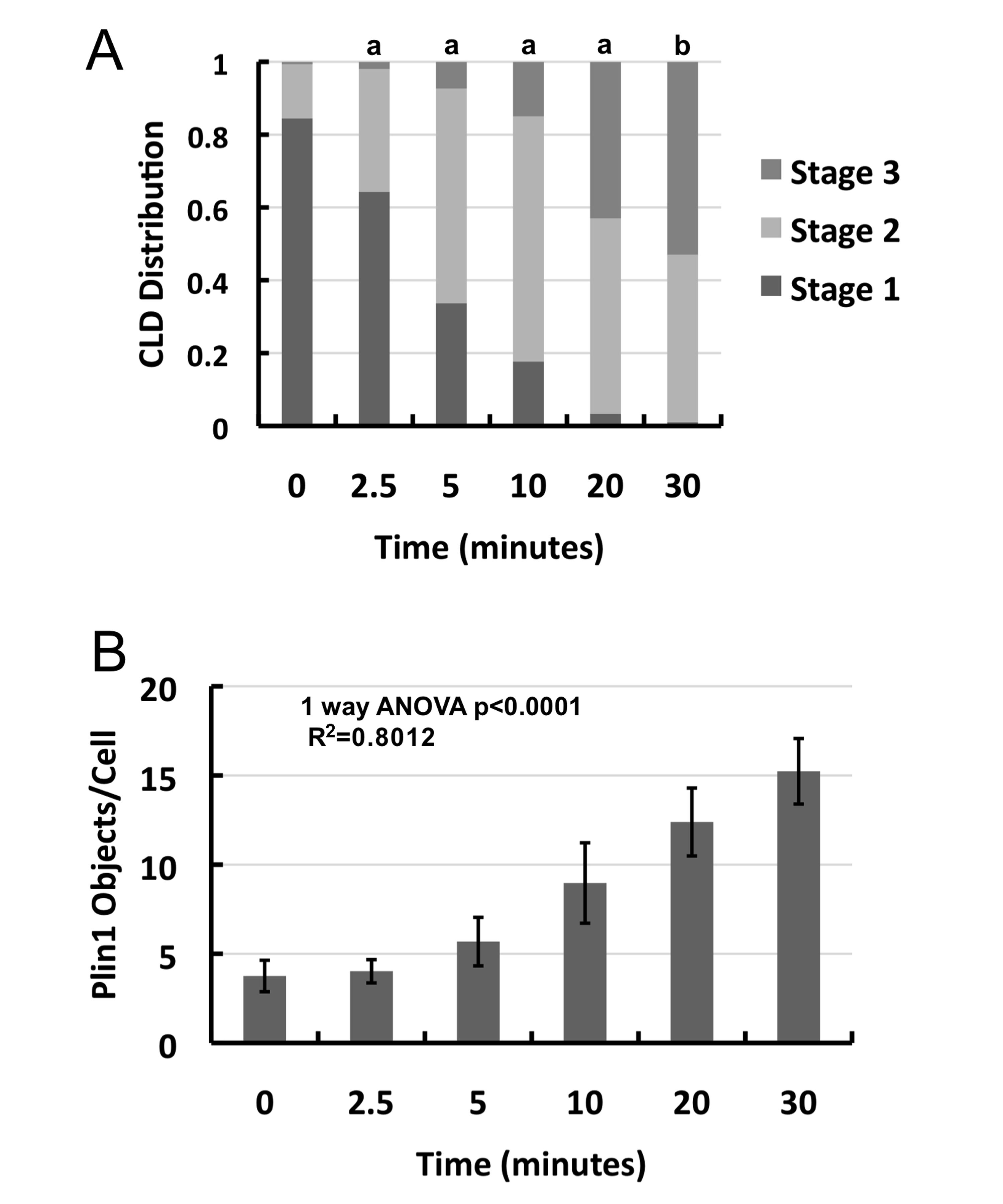

Supplement: Figure S5 — Forskolin stimulated cluster dispersion. (A) The change in CLD clustering as a function of time after exposure to 10 µM forskolin monitored by morphological analysis. The values are averages of 3 experiments, in each experiment 60–100 cells were assayed per time point. Statistical significance is indicated by lower case letters a and b above the bars: a, stage values are different from values at previous time points (p<0.001); b, values are different from 0, 2.5, 5, and 10 minute values (p<0.001). (B) CLD dispersion is determined as a function of time after exposure to 10 µM forskolin by quantifying Plin1 objects/cell. Values are means ± SEM for 3 experiments again examining 60–100 cells per time point in each experiment. A 1-way ANOVA analysis of Plin1 objects/cell yields a p<0.0001; the post test for linear trends p<0.0001, and R2 = 0.0.8012 (TIF) [file pone.0066837.s005.tif]

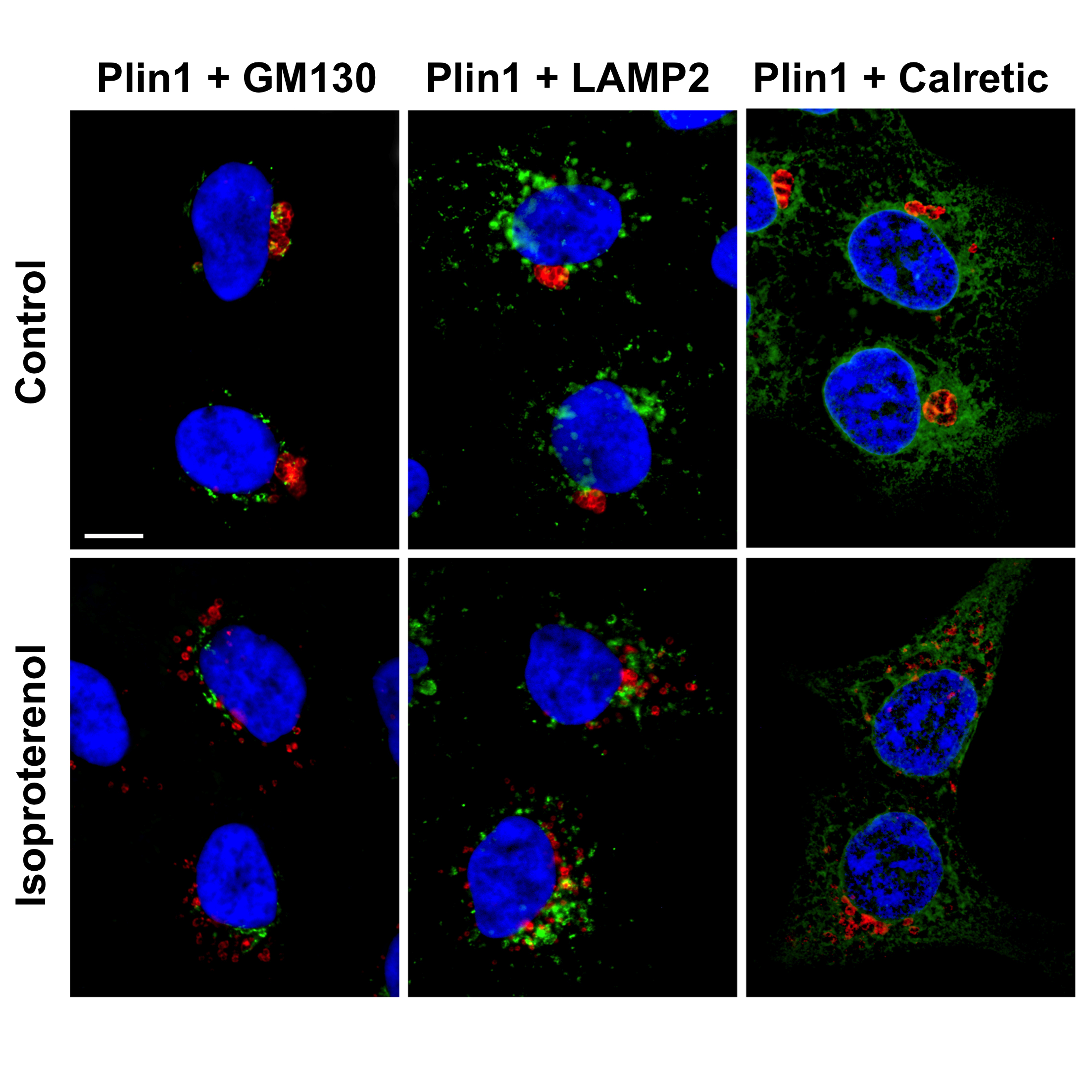

Supplement: Figure S6 — Plin1-coated clustered CLD do not localize near Golgi, lysosomes, or endoplasmic reticular structures. Representative immunofluorescence images of Plin1 (red) and: Golgi membrane protein 130 (GM130, green); lysosomal membrane associated protein 2 (LAMP2, green); the endoplasmic reticulum protein calreticulin (Calretic, green) in Plin1 cells treated with vehicle (Control) or 10 µg/ml isoproterenol for 1 hour (Isoproterenol). Hoechst-stained nuclei are shown in blue. The size bar is 10 µm. (TIF) [file pone.0066837.s006.tif]

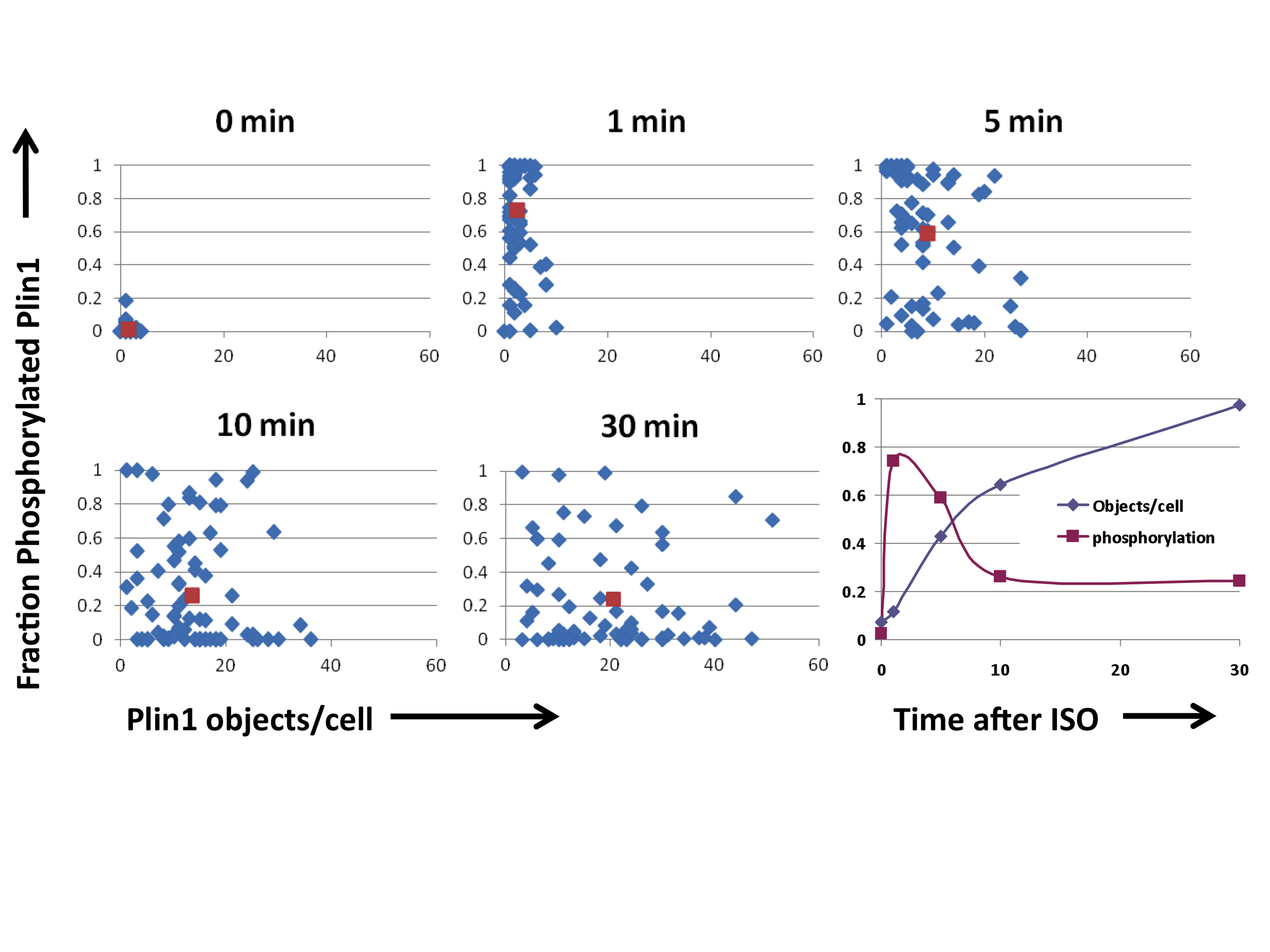

Supplement: Figure S8 — Time course single cell analysis of Plin1-S492 phosphorylation following isoproterenol stimulation. The data show the results of single cell analyses comparing the relative amounts of Plin1 phosphorylated on S492 and the extent of CLD dispersion, as determined from the number Plin1 objects/cell, at time points 0, 1, 5, 10, and 30 minutes. Approximately 50–75 cells per time point are shown. The results from individual cells are represented by a blue diamond, cell averages are represented by red squares. Also shown are graphs for the mean fraction Plin1-S492-phosphorylated and mean dispersion at these same time points following isoproterenol induced dispersion. (TIF) [file pone.0066837.s008.tif]
